# Shape Transitions of Red Blood Cell under Oscillatory Flows in Microchannels

**DOI:** 10.21203/rs.3.rs-3296659/v1

**Published:** 2023-08-30

**Authors:** Lahcen Akerkouch, Trung Le

**Affiliations:** 1 Department of Civil, Construction, and Environmental Engineering, North Dakota State University, 1410 14th N, Fargo, 58102, ND, USA.

**Keywords:** Red Blood Cell, Dissipative Particle Dynamics, Immersed Boundary Method, Oscillatory flow

## Abstract

This paper aims to examine the ability to control Red Blood Cell (RBCs) dynamics and the associated extracellular flow patterns in microfluidic channels via oscillatory flows. Our computational approach employs a hybrid continuum-particle coupling, in which the cell membrane and cytosol fluid are modeled using the Dissipative Particle Dynamics (DPD) method. The blood plasma is modeled as an incompressible fluid via the Immersed Boundary Method (IBM). This coupling is novel because it provides an accurate description of RBC dynamics while the extracellular flow patterns around the RBCs are also captured in detail. Our coupling methodology is validated with available experimental and computational data in the literature and shows excellent agreement. We explore the controlling regimes by varying the shape of the oscillatory flow waveform at the channel inlet. Our simulation results show that a host of RBC morphological dynamics emerges depending on the channel geometry, the incoming flow waveform, and the RBC initial location. Complex dynamics of RBC are induced by the flow waveform. Our results show that the RBC shape is strongly dependent on its initial location. Our results suggest that the controlling of oscillatory flows can be used to induce specific morphological shapes of RBCs and the surrounding fluid patterns in bio-engineering applications.

## 1 Introduction

Extensive research has been conducted in the last few decades on the morphological change of Red Blood Cells (RBCs) in fluid flows due to their importance in blood pathology (Kaul et al, 1983; Barabino et al, 2010; Secomb, 2017). It has been shown that the response of the RBC membrane to blood plasma dynamics can affect the overall patterns of microvascular blood flows (Tomaiulo et al, 2009; Guckenberger et al, 2018; Reichel et al, 2019). Despite a substantial body of literature, the dynamics of RBCs remain a significant challenge to be studied due to the complexity of various response modes, which result from the interaction of the suspended cellular membrane with shear flow (Vlahovska et al, 2011). Several factors can affect the dynamics of RBCs, such as stiffness of the membrane (Czaja et al, 2020), shear rate $\left(\overset{-}{\dot{\gamma }}\right)$ (Lanotte et al, 2016; Mauer et al, 2018), and viscosity contrast (λ) between the blood plasma and the cytosol (Mauer et al, 2018), among other factors. As a result, the RBC deformation process in shear flow is not well understood, especially under time-dependent shear rates (Schmidt et al, 2022; Krauss et al, 2022).

In free shear flows with a constant shear rate $\left({\overset{-}{\dot{\gamma }}}_{0}\right)$, the shear strength is the controlling parameter of the RBC dynamics, in which the shape of RBCs becomes increasingly complex (more lobes) as the shear rate increases (Dupire et al, 2012). In particular, for the range of shear rate $\left({\overset{-}{\dot{\gamma }}}_{0}\right)$ from $10{\;s}^{-1}$ to $2,000\;s^{-1}$, the dynamics of RBCs can be classified into three main regions (Lanotte et al, 2016): (*i*) tumbling at weak shear rate $\left({\overset{-}{\dot{\gamma }}}_{0}<10{\;s}^{-1}\right)$; (*ii*) circular/elliptical stomatocytes $\left(10{\;s}^{-1}<{\overset{-}{\dot{\gamma }}}_{0}<400{\;s}^{-1}\right)$; and (*iii*) multilobes $\left(400s^{-1}<{\overset{-}{\dot{\gamma }}}_{0}<2,000\;s^{-1}\right)$. In the tumbling region, the RBC deformation is minimal and reversible, which allows the RBCs to maintain their biconcave discoid shape. As the shear rate increases to $400{\;s}^{-1}$, the percentage of discocytes decreases and stomatocytes start to dominate. The rolling and tumbling stomatocytes appear at ${\overset{-}{\dot{\gamma }}}_{0}=150{\;s}^{-1}$ and $250{\;s}^{-1}$, respectively. This pattern persists up until ${\overset{-}{\dot{\gamma }}}_{0}=400{\;s}^{-1}$ when the stomatocytes assume a shape with an elliptical rim. In the range $400{\;s}^{-1}<{\overset{-}{\dot{\gamma }}}_{0}<2,000{\;s}^{-1}$, RBCs with large lobes on their surface, which are referred to as trilobes or hexalobes, emerge.

Studies of RBC dynamics in microchannels have shown that the RBC can transition from its biconcave discoid shape to different morphologies (Noguchi† and Gompper, 2005; Fedosov et al, 2014; Guckenberger et al, 2018) under specific combinations (state diagram) of viscosity contrast, shear rate (the capillary number - $C_{a}$) (Kaoui et al, 2011a) and channel confinement (χ) (Mauer et al, 2018; Fedosov et al, 2014; Reichel et al, 2019). The state diagram has revealed two main categories of RBC morphological shapes: (*i*) symmetrical; and (*ii*) asymmetrical types (Tomaiulo et al, 2009; Quint et al, 2017; Kihm et al, 2018). The simple type contains three modes (Coupier et al, 2012): (*a*) bullet; (*b*) croissant (in rectangular channels); and (*c*) parachute (in circular channels) shapes, while the complex type includes (Kaoui et al, 2009; Reichel et al, 2019): (*a*) slipper; (*b*) multilobes; (*c*) trilobes; and (*d*) hexalobes shapes. The shape transition in the simple type has been shown to reach a terminal shape (either bullet, croissant, or parachute). However, it is still not fully clear whether or not the complex shapes are stable or they are just transient states (Noguchi† and Gompper, 2005; Kaoui et al, 2011b). In the complex shape mode (Kaoui et al, 2009), the shape transition mostly depends on the flow lag, which is the difference between the translation velocity of the RBC and the velocity of blood plasma. In brief, it is unclear how the complex shapes emerge from the biconcave discoid shape.

Two shapes are the most frequently observed (Guckenberger et al, 2018): (*i*) the croissant shape (symmetrical); and (*ii*) the slipper shape (asymmetrical). In particular, the slipper shape is characterized by the tank-treading motion of the cell membrane, which is essentially a self-rotation of the membrane around its center of mass during the RBC propagation (McWhirter et al, 2009; Guckenberger et al, 2018). Experimental and computational studies have shown that these morphological shapes might result in distinct flow structures of blood plasma in the vicinity of the RBC (Guckenberger et al, 2018). For instance, there exists a closed vortex downstream of the RBC when the slipper shape emerges (Yaya et al, 2021). Such a vortex is absent during the croissant shape. To our knowledge, there has been no systematic effort in understanding the emergence of the extracellular flow patterns as the morphological shape of the RBC changes.

Recently, oscillatory flow (time-dependent shear rate - ${\overset{-}{\dot{\gamma }}}_{f}$) has been shown to be a promising technique for cell separation. Because cell deformation is irreversible under time-dependent shear rates (Mutlu et al, 2018; Schmidt et al, 2022), oscillatory flows have been suggested to sort RBCs based on their size and deformability (Krauss et al, 2022). Oscillatory flows can reduce the required travel distance of cells because it induces the lateral migration of cells in a short axial distance. This feature simplifies the design of microfluidic channels and thus improves the cell separation process (Lafzi et al, 2020). However, it is unclear on the process of morphological transition as the RBC responds to the time-dependent shear rate $\left({\overset{-}{\dot{\gamma }}}_{f}\right)$ during this lateral migration. Therefore, it is necessary to investigate this process in detail.

In this work, we utilized our hybrid continuum-particle simulation methodology (Akerkouch and Le, 2021) to study the response of the RBC to time-dependent shear rates. Our paper is organized as follows. First, a brief description of the numerical methods for simulating the blood plasma and the RBC is presented. Second, the obtained RBC dynamics are validated with experimental data under (i) stretching force; (*ii*) constant shear rates (croissant and slipper shapes); (*iii*) oscillatory shear rates. Third, we perform a parametric study where the shear rate waveform, the peak flow rate, and the initial position of the RBC are varied to induce a host of RBC morphological changes. Finally, the relationship between the RBC’s shape and the extracellular flow patterns is reported as a basis for cell manipulation in future applications.

## 2 Methodology

### 2.1 The idealized shape of the RBC

**Fig. 1:**
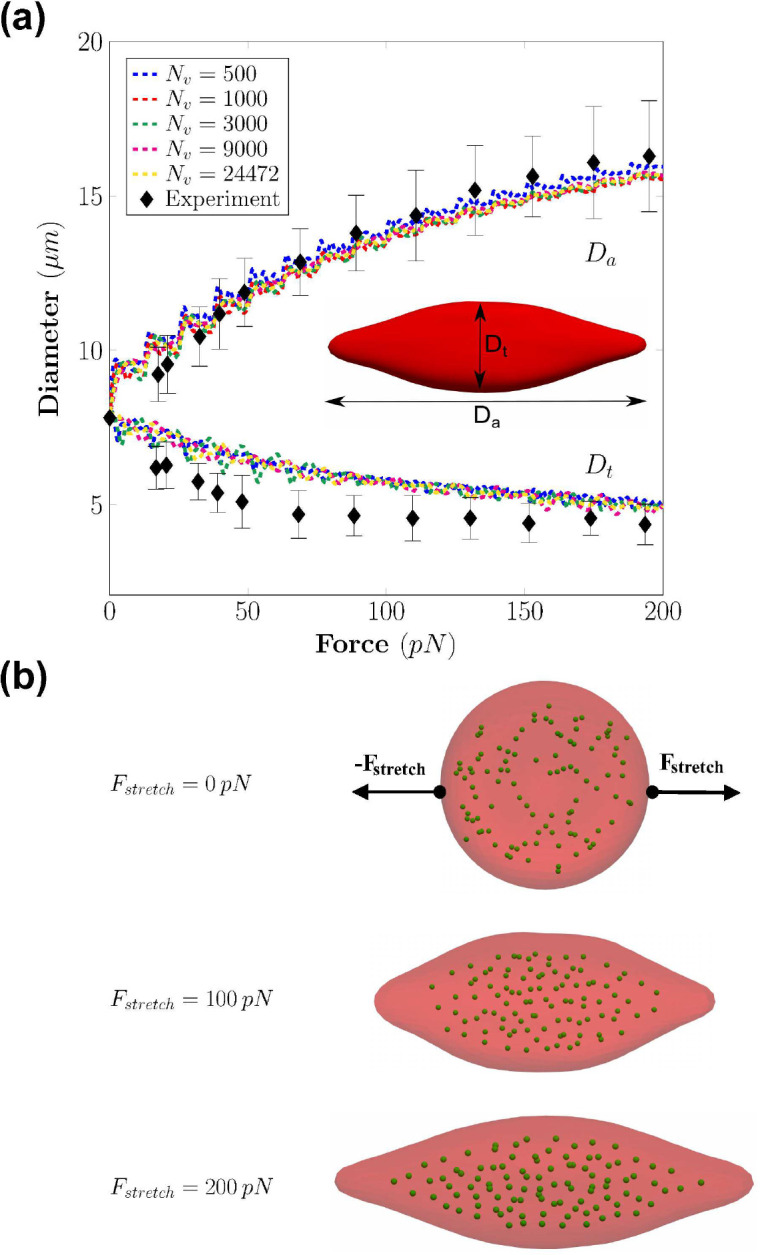
(*a*) The recorded axial $\left(D_{a}\right)$ and transverse $\left(D_{t}\right)$ diameters (dash lines) of the RBC under incremental stretching at different coarse-graining levels $\left(N_{v}\right)$. The experimental data of Mills et al (2004) are shown as black diamonds. (*b*) The deformed shapes of the RBC membrane and the cytoplasm (green particles) under different stretching forces $F_{\mathrm{stretch}}$.

The idealized shape of the RBC membrane is given by a set of points with coordinates (x,y,z) in 3D space with the analytical equation (Fedosov et al, 2010) as shown in Figure 1:

(1) $$z=\pm D_{0}\sqrt{1-\frac{4\left(x^{2}\;+\;y^{2}\right)}{D_{0}^{2}}}\left[a_{0}\;+\;a_{1}\frac{x^{2}\;+\;y^{2}}{D_{0}^{2}}\;+\;a_{2}\frac{{\left(x^{2}\;+\;y^{2}\right)}^{2}}{D_{0}^{4}}\right],$$

the parameters are chosen in this work as $D_{0}=7.82\;\mu m$ (the equilibrium diameter), $a_{0}=0.00518$, $a_{1}=2.0026$, and $a_{2}=-4.491$. Note that the idealized shape will be used as the initial shape of the RBC membrane for all simulation cases. The membrane mechanics that govern the cellular deformation under loadings will be described in the following sections.

### 2.2 RBC membrane model

As the idealized surface of the RBC membrane is known precisely according to Equation (1), a triangulation procedure is carried out to mimic the distribution of the spectrin links on the membrane as edges of each triangular element (links) (Fedosov et al, 2010). A network of non-linear springs is generated for each edge to model the dynamics of the spectrin links (Pivkin and Karniadakis, 2008; Fedosov et al, 2010; Akerkouch and Le, 2021). At each vertex i, the dynamics of the links are determined by the membrane force $F_{i}^{membrane}$, which is linked to Helmholtz’s free energy $V_{i}$ at the same vertex i through the following relationship:

(2) $$F_{i}^{membrane}=-\frac{\partial V_{i}}{\partial r_{i}},$$

with $r_{i}$ is the position vector of the vertex i.

The potential $V\left(\left\{r_{i}\right\}\right)$ incorporates the physical properties of the lipid bilayer: (*a*) in-plane stretching; (*b*) bending stiffness; (*c*) area and volume conservation; and (*d*) membrane viscosity

(3) $$V\left(\left\{r_{i}\right\}\right)=V_{in-plane}+V_{bending}+V_{area}+V_{volume}$$

#### 2.2.1 Action potential models

The in-plane free energy term $V_{in-plane}$ includes the elastic energy stored in the membrane modeled using the nonlinear Wormlike Chain and Power (WLC-POW) spring model. Here, the WLC-POW potential is computed for each link j formed by two vertices as,

(4) $$V_{in-plane\;}=\sum_{j\in 1\ldots N_{s}}\left[U_{WLC}\left(l_{j}\right)+U_{POW}\left(l_{j}\right)\right],$$

with $N_{s}$ as the total number of links forming the triangulated mesh.

The WLC attractive potentials $U_{WLC}\left(l_{j}\right)$ for individual link $l_{j}$ is expressed as:

(5) $$U_{WLC}=\frac{k_{B}Tl_{max}}{4p}\frac{3x^{2}\;-\;2x^{3}}{1\;-\;x},$$

where the value $x=\frac{l_{j}}{l_{max}}$ represents the spring deformation, in which $l_{j}$, $l_{max}$, p, $k_{B}$, and T are the length of the spring j, the maximum allowable length for the links, the persistence length, Boltzmann’s constant, and the temperature, respectively.

The repulsive force, described by the energy potential $U_{POW}\left(l_{j}\right)$, takes the form of a power function (POW). The separation distance $l_{j}$ is a determining factor in the calculation of $U_{POW}$, which is given by:

(6) $$U_{POW}\left(l_{j}\right)=\frac{k_{p}}{(m\;-\;1)l_{j}^{m-1}}\;m>0\;\text{and}\;m\neq 1,$$

where $k_{p}$ is the POW force coefficient. In this work, the value m=2 is used for the exponent (Fedosov et al, 2010).

The bending energy $V_{bending}$ accounting for the membrane resistance to bending is defined as,

(7) $$V_{bending}=\sum_{j\in 1\ldots N_{s}}k_{b}\left[1-cos\left(\theta_{j}-\theta_{0}\right)\right],$$

with $k_{b}$, $\theta_{0}$ and $\theta_{j}$ are the bending rigidity, the spontaneous angle and the instantaneous angle between the normal vectors of two adjacent triangles sharing a common edge (link) j, respectively.

The area and volume conservation constraints account for the incompressibility of the lipid bilayer and the inner cytosol, respectively. They are defined as:

(8) $$\begin{array}{l} V_{area}=\frac{k_{a}{\left(A\;-\;A_{0}^{tot}\right)}^{2}}{2A_{0}^{tot}}+\sum_{k\in 1\ldots N_{t}}\frac{k_{d}\left(A_{k}\;-\;A_{0}\right)}{2A_{0}},\\ V_{volume}=\frac{k_{v}{\left(V\;-\;V_{0}^{tot}\right)}^{2}}{2V_{0}^{tot}}, \end{array}$$

with $N_{t}$ as the total number of triangles. $k_{a}$, $k_{d}$, and $k_{v}$ are the global area, local area and volume constraint coefficients, respectively. $A_{k}$ and $A_{0}$ are the instantaneous area of the *k*^*th*^ triangle (element) and the initial value of the average area per element. $A_{0}^{tot}$ and $V_{0}^{tot}$ are the RBC’s equilibrium total area and volume, respectively. A and V are the instantaneous total area and total volume of the RBC. The detailed procedure to evaluate the values of A and V for individual elements was reported in our previous work (Akerkouch and Le, 2021).

Equation (2) is used to calculate the precise nodal forces for each potential energy V in Equations (4) - (8) (Akerkouch and Le, 2021; Fedosov et al, 2010). The internal force $F_{i}^{membrane}$ contribution from *i*^*th*^ vertex can be computed by summing all the nodal forces as:

(9) $$F_{i}^{membrane}=F_{i}^{WLC}+F_{i}^{POW}+F_{i}^{Bending}+F_{i}^{{Area}_{g}}+F_{i}^{{Area}_{loc}}+F_{i}^{Volume}.$$

#### 2.2.2 Cellular membrane/cytoskeleton interaction

To account for the interaction between the cytoskeleton and the lipid bilayer, the bilayer-cytoskeletal interactions force $F^{E}$ is incorporated into the total RBC membrane forces (Peng et al, 2013a). The membrane thickness of a healthy RBC is typically 10 *nm*, composed of an outer layer (lipid bilayer) and the inner layer (skeleton) (Li et al, 2018). In this study, $F^{E}$ is applied when the distance between two elements with opposite normal vectors is at least 20 times the thickness of the membrane. The force $F^{E}$ is applied equally to all the vertices (i=1,2and3) for each of the two elements. The bilayer-cytoskeletal interactions force is given by:

(10) $$F_{i}^{E}=k_{bs}\;n,$$

with the stiffness of the bilayer-cytoskeletal $\left(k_{bs}=4.12\;pN/\mu m\right)$ is assumed to be in the same order as one of the membrane spectrin networks (Peng et al, 2013a). n is the normal vector of the triangle.

### 2.3 Modeling membrane-cytosol interactions

The interaction between the membrane and the cytosol is modeled using the Dissipative Particles Dynamics (DPD method). DPD is a microscopic simulation technique widely used to model the flow of complex fluids. Here, the flow is described as a group of clustered interacting particles according to the Lagrangian approach (Fedosov et al, 2010). In this work, the cytosol within the RBC is modeled using a set of randomly distributed DPD particles $\left(N_{f}\right)$ that fill the internal volume of the cell (Pivkin and Karniadakis, 2008; Fedosov et al, 2010) as shown in Figure 1.

Due to the different nature of the interactions, the component of the total force of each particle $F_{i}$ depends on the nature of the particle i (either membrane or cytosol particle). In general, each DPD particle i interact with surrounding particles j within a cut-off radius $r_{c}$ through three pairwise additive forces: (*a*) the conservative force $F_{ij}^{C}$; (*b*) the dissipative force $F_{ij}^{D}$; and (*c*) the random force $F_{ij}^{R}$. The relative position vector between the particles i and j and related terms are given by: $r_{ij}=r_{i}-r_{j}$, the distance $r_{ij}=\left|r_{ij}\right|$, and the unit vector ${\overset{ˆ}{r}}_{ij}=\frac{r_{ij}}{r_{ij}}$. Also, $v_{i,j}=v_{i}-v_{j}$ is the relative velocity between the particles i and j with velocities $v_{i}$ and $v_{j}$.

For a DPD particle i of the cytosol fluid, the total force $F_{i}$ is:

(11) $$F_{i}=\sum_{j\neq i}F_{ij}^{C}+F_{ij}^{D}+F_{ij}^{R}.$$

For membrane particles, the total force $F_{i}$ acting on each membrane particle is given by the sum of the membrane force $F_{i}^{membrane}$, the bilayer-cytoskeletal interactions force $F^{E}$ and the contributing forces from the surrounding DPD fluid particles (the cytosol):

(12) $$F_{i}=F_{i}^{membrane}+F_{i}^{E}+\sum_{j\neq i}\;F_{ij}^{C}+F_{ij}^{D}+F_{ij}^{R}.$$

The mathematical formulation of the conservative force $F_{ij}^{C}$, the dissipative force $F_{ij}^{D}$, and the random force $F_{ij}^{R}$ for the membrane and the cytosol fluid particles are explained below.

#### 2.3.1 The conservative force

The conservative force $F_{ij}^{C}$ is given by:

(13) $$\begin{array}{l} \;F_{ij}^{C}\;=F_{ij}^{C}\left(r_{ij}\right){\overset{ˆ}{r}}_{ij},\\ F^{C}\left(r_{ij}\right)\;=\left\{\begin{array}{ll} a_{ij}\left(1-\frac{r_{ij}}{r_{c}}\right) & \text{for}\;r_{ij}\leq r_{c}\\ 0 & \text{for}\;r_{ij}>r_{c,} \end{array}\right. \end{array},$$

where $a_{ij}=20$ is the conservative force coefficient between particles i and j. Note that the particles i and j can be either membrane or cytosol fluid particles. Thus, there are two types of interactions: i) cytosol fluid/fluid; and ii) membrane/fluid-particle interactions (Fedosov et al, 2010).

#### 2.3.2 The dissipative force

The dissipative force $F_{ij}^{D}$ for the membrane particles is computed as:

(14) $$F_{ij}^{D}=-\Gamma^{T}v_{ij}-\Gamma^{C}\left(v_{ij}\cdot {\overset{ˆ}{r}}_{ij}\right){\overset{ˆ}{r}}_{ij}.$$

The membrane viscosity is a function of both dissipative parameters, $\Gamma^{T}$ and $\Gamma^{C}$. The superscripts T and C denote the translational and central components. Here, $\Gamma^{T}$ is responsible for a large portion of the membrane viscosity in comparison to $\Gamma^{C}$. In addition, $\Gamma^{C}$ is assumed to be equal to one-third of $\Gamma^{T}$ in Equations (14)(Fedosov et al, 2010). Consequently, these parameters relate to the physical viscosity of the membrane $\eta_{m}$ as:

(15) $$\left\{\begin{array}{l} \eta_{m}=\sqrt{3}\Gamma^{T}+\frac{\sqrt{3}\Gamma^{C}}{4},\\ \;\Gamma^{C}=\frac{\Gamma^{T}}{3}. \end{array}\right.$$

Hence, the dissipative force $F_{ij}^{D}$ among the membrane particles can be expressed as:

(16) $$F_{ij}^{D}=-\frac{12}{13\sqrt{3}}\eta_{m}v_{ij}-\frac{4}{13\sqrt{3}}\eta_{m}\left(v_{ij}\cdot {\overset{ˆ}{r}}_{ij}\right){\overset{ˆ}{r}}_{ij}.$$

The dissipative force $F_{ij}^{D}$ for the cytosol fluid particles is defined as:

(17) $$F_{ij}^{D}=-\gamma \omega^{D}\left(r_{ij}\right)\left(v_{ij}\cdot {\overset{ˆ}{r}}_{ij}\right){\overset{ˆ}{r}}_{ij},$$

the quantity γ is a constant coefficient defining the strength of the dissipative force. The weight functions, $\omega^{D}\left(r_{ij}\right)$ and $\omega^{R}\left(r_{ij}\right)$ are given by:

(18) $$\omega^{D}\left(r_{ij}\right)={\left[\omega^{R}\left(r_{ij}\right)\right]}^{2},$$

(19) $$\omega^{R}\left(r_{ij}\right)=\left\{\begin{array}{ll} {\left(1-\frac{r_{ij}}{r_{c}}\right)}^{s} & \text{for}\;r_{ij}\leq r_{c},\\ 0 & \text{for}\;r_{ij}>r_{c}, \end{array}\right.$$

with s=1 following the original DPD method (Fedosov et al, 2010). The particle i represents the fluid particle, while the particle j can be either a fluid or membrane particle within the cut-off radius $r_{c}$.

#### 2.3.3 The random force

Using the assumptions in Equation (15), the random force for membrane particles can be described as:

(20) $$F_{ij}^{R}=\sqrt{2k_{B}T}\left(2\sqrt{\frac{2\sqrt{3}}{13}\eta_{m}}d\overset{-}{W_{ij}^{S}}\right){\overset{ˆ}{r}}_{ij},$$

where $tr\left(dW_{ij}\right)$ is the trace of the random matrix of independent Wiener increments $dW_{ij}$, and $d\overset{-}{W_{ij}^{S}}=dW_{ij}^{S}-\frac{tr\left(dW_{ij}^{S}\right)}{3}$ is the traceless symmetric part.

The random force $F_{ij}^{R}$ for the cytosol fluid are defined as:

(21) $$F_{ij}^{R}=\sigma \omega^{R}\left(r_{ij}\right)\cdot \frac{\vartheta_{ij}}{\sqrt{dt}}\cdot {\overset{ˆ}{r}}_{ij},\;\sigma^{2}=2\gamma k_{B}T,$$

where σ is a constant coefficient defining the strength of the random force, dt is the physical time step, ϑ is a normally distributed random variable with zero mean and unit variance and $\vartheta_{ij}=\vartheta_{ji}$. Note that the particles i and j must be both cytosol fluid particles.

#### 2.3.4 Plasma and cytosol viscosity contrast

At the physiological condition, the viscosity ratio between the blood plasma and the RBC cytosol is equal to 5.0 $\left(\lambda =\frac{\mu_{cytosol}}{\mu_{plasma}}=5.0\right)$ (Wells and Schmid-Schönbein, 1969). To ensure that this condition is met, the dynamic viscosity of the plasma is set to be $\mu_{plasma}=1.2\;mPa\;s$. The viscosity condition is enforced on the cytosol fluid by calibrating the parameters of the dissipative and the random forces (Mai-Duy et al, 2020) (γ and σ). Specifically, the dynamic properties of the DPD particles of the cytosol fluid are given in the dimensionless DPD unit as (Fan et al, 2006):

(22) $$\begin{array}{r} \text{mass}\;\text{diffusivity:}\;D_{f}=\frac{45k_{B}T}{2\pi \gamma \rho r_{c}^{3}},\\ \text{dynamics}\;\text{viscosity:}\;\mu =\frac{\rho D_{f}}{2}+\frac{2\pi \gamma \rho^{2}r_{c}^{5}}{1575}, \end{array}$$

with ρ as the density. The DPD dimensionless parameters and physical units are linked (Ghoufi et al, 2013) in order to compute the coefficients of the dissipative and randoms forces for the cytosol dynamic viscosity of $\mu_{cytosol}=6\;mPa\;s$. The details of the conversion procedure are summarized in Table 1.

### 2.4 Scaling of model and physical units

One challenge in DPD modeling is establishing a relationship between the modeled quantities and the physical values (Ye et al, 2019). Since this relationship is not explicit, it is necessary to use a scaling argument to recover this relationship (Fedosov et al, 2010; Peng et al, 2013b). For each parameter, the superscript M and P correspond to the model and physical units, respectively. The details of the physical parameters for the RBC are shown in the Tables 2.

The length scale $r^{M}$ is defined as:

(23) $$r^{M}=\frac{D_{0}^{P}}{D_{0}^{M}}(m),$$

The energy per unit mass $k_{B}T$ and the force N scaling values are given by:

(24) $$\begin{array}{l} {\left(k_{B}T\right)}^{M}\;=\frac{Y^{P}}{Y^{M}}{\left(\frac{D_{0}^{P}}{D_{0}^{M}}\right)}^{2}{\left(k_{B}T\right)}^{P},\\ N^{M}\;=\frac{Y^{P}}{Y^{M}}\frac{D_{0}^{P}}{D_{0}^{M}}N^{P}, \end{array}$$

with Y is the membrane Young’s modulus.

The timescale τ is defined as follows:

(25) $$\tau ={\left(\frac{D_{0}^{P}}{D_{0}^{M}}\frac{\eta_{m}^{P}}{\eta_{m}^{M}}\frac{Y^{M}}{Y^{P}}\right)}^{\alpha },$$

with α=1 is the scaling exponent.

### 2.5 Coarse-graining procedure

A full-scale model of the RBC typically consists of millions of particles, which are required to accurately simulate protein dynamics (Tang et al, 2017). However, it is not feasible to use such a full-scale model in a Fluid-Structure Interaction (FSI) simulation due to the high computational cost. We followed the coarse-graining procedure of Pivkin and Karniadakis (2008) to represent the RBC membrane by a smaller number of particles (coarse-grained model). This procedure does not allow a detailed simulation of separate proteins, but it is versatile enough to capture the overall dynamics of the RBC membrane. The parameters of the coarse-grained model (c) are computed from the ones of the fine-scaled model (f) by a scaling procedure. Examples of such parameters are explained below.

Based on the equilibrium condition, Pivkin and Karniadakis (2008) proposed a coarse-graining procedure based on the area/volume constraint for the spring equilibrium $l_{0}$ and maximum $l_{max}$ lengths as follows:

(26) $$l_{0}^{c}=l_{0}^{f}\sqrt{\frac{N_{v}^{f}\;-\;2}{N_{v}^{c}\;-\;2}}\;\;\text{and}\;\;l_{max}^{c}=l_{max}^{f}\sqrt{\frac{N_{v}^{f}\;-\;2}{N_{v}^{c}\;-\;2}},$$

the role of $l_{0}$ and $l_{max}$ is critical in determining the response from the WLC model as seen in Equation (5), with $l_{max}=2.2\;l_{0}$ in the fine-scaled model. Due to the scaling in Equation (26), the value of $x_{0}=\frac{l_{0}}{l_{max}}=\frac{1}{2.2}$ does not change as the model is coarse-grained from the number of vertices $N_{v}^{f}$ to $N_{v}^{c}$. In this work, a range of $N_{v}^{c}$ values are considered as shown in Table 3 with $N_{v}^{f}$ set to be 27, 472.

Furthermore, as the number of vertices reduces, the average angle between the pairs of adjacent triangles increases. Therefore, the spontaneous angle θ is adjusted accordingly in the coarse-grained model as:

(27) $$\theta_{0}^{c}=\theta_{0}^{f}\frac{N_{v}^{f}}{N_{v}^{c}}\;\text{with}\;\theta_{0}^{f}=\arccos\left(\frac{\sqrt{3}(N_{v}^{f}\;-\;2)\;-\;5\pi }{\sqrt{3}(N_{v}^{f}\;-\;2)\;-\;3\pi }\right).$$

To maintain the shear and area-compression moduli, the parameters p and $k_{p}$ are adjusted as:

(28) $$p^{c}=p^{f}\frac{l_{0}^{f}}{l_{0}^{c}}\;\;\text{and}\;\;k_{p}^{c}=k_{p}^{f}{\left(\frac{l_{0}^{c}}{l_{0}^{f}}\right)}^{m+1}.$$

The details of the parameters for the coarse-graining process are shown in Table 3.

### 2.6. Time integration

We implemented the modified Velocity-Verlet algorithm (Groot and Warren, 1997), which consists of two primary steps. The first step involves determining the new position of the particle $i\left(r_{i}\right)$ while predicting the velocity $\left({\tilde{v}}_{i}\right)$, and the second step involves correcting the velocity by utilizing the computed force $\left(F_{i}\right)$ based on the predicted velocity and the new position as follows.

(29) $$\begin{array}{l} r_{i}(t+dt)\;=r_{i}(t)+dtv_{i}(t)+\frac{1}{2}dt^{2}F_{i}(t),\\ {\tilde{v}}_{i}(t+dt)\;=v_{i}(t)+\Lambda dtF_{i}(t),\\ F_{i}(t+dt)\;=F_{i}\left(r_{i}(t+dt),{\tilde{v}}_{i}(t+dt)\right),\\ v_{i}(t+dt)\;=v_{i}(t)+\frac{1}{2}dt\left(F_{i}(t)+F_{i}(t+dt)\right), \end{array}$$

where ${\tilde{v}}_{i}(t+dt)$ is the predictive velocity at time t+dt and Λ is the variable which accounts for the effects of the stochastic processes. The value of Λ is chosen to be the optimal value (Groot and Warren, 1997) Λ=0.65.

### 2.7 Fluid-Structure Interaction simulation of RBC in flows

The blood plasma is considered as an incompressible Newtonian fluid modeled using the incompressible three-dimensional unsteady Navier-Stockes equations, with density ρ and kinematic viscosity $\nu =\frac{\mu_{plasma}}{\rho }$. The governing equations (continuity and momentum) are read in Cartesian tensor notation as follows (i=1,2,3 and repeated indices imply summation):

(30) $$\frac{\partial u_{i}}{\partial x_{i}}=0,$$

(31) $$\frac{\partial u_{i}}{\partial t}+\frac{\partial \left(u_{i}u_{j}\right)}{\partial x_{j}}=-\frac{\partial p}{\partial x_{i}}+\nu \frac{\partial^{2}u_{i}}{\partial x_{j}\partial x_{j}}.\;$$

In the above equations, $u_{i}$ is the *i*^*th*^ component of the velocity vector u; t is time; $x_{i}$ is the *i*^*th*^ spatial coordinate; p is the pressure divided by ρ. The characteristic velocity scale is chosen as $U_{0}$. The length scale $L_{s}$ is set to equal 8 *μm* for all cases. Note that this length scale is chosen to reflect the diameter of the RBC at the equilibrium condition $\left(L_{s}\approx D_{0}\right)$.

The fluid solver is based on the sharp-interface curvilinear-immersed boundary (CURVIB) method in a background curvilinear domain that contains the RBC model (Ge and Sotiropoulos, 2007). The CURVIB method used here has been applied and validated in various FSI problems across different biological engineering areas (Le et al, 2010; Le and Sotiropoulos, 2012; Le et al, 2019). In our previous work (Akerkouch and Le, 2021), we utilize the capabilities of the CURVIB method to capture accurately the complex cellular dynamics of the RBC in fluid flows.

### 2.8 Computational setups

**Fig. 2:**
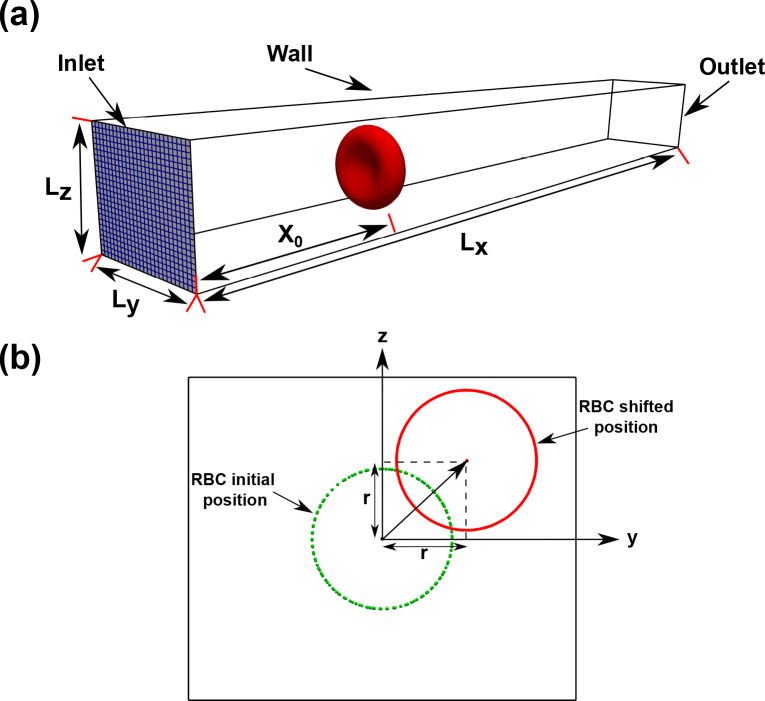
(*a*) The computational setup for the FSI simulation of a single RBC in a rectangular channel of size $L_{x}\times L_{y}\times L_{z}$. The inlet plane is shown in blue, which shows uniform grid lines to illustrate the computational mesh. The RRBC is placed at an axial distance $x_{0}$ from the inlet plane. (*b*) The sketch of the cross-section of the computational domain to illustrate the definition of the radial shift (r). The dashed line shows that the RBC is placed at the channel’s center line. The solid line depicts how the cell is transversely shifted from the cross-sectional center along the bisector of the first quadrant by a radial shift (r) in the y-z plane (Table 7).

Fluid-Structure Interaction simulations are performed to determine the dynamics of RBC in a confined micro-channel (Guckenberger et al, 2018). The computation domain is defined as a rectangular channel containing a single RBC as illustrated in Figure 2a. The dimensions of the domain along the x, y, and z are $L_{x}$ (the length), $L_{y}$ (the width) and $L_{z}$ (the height), respectively. The computational domain is discretized as a structured grid of size $N_{i}\times N_{j}\times N_{k}$ with the spatial resolution in three directions (i,j,k) are Δx×Δy×Δz, respectively. The details of the channels used in the simulations are listed in Table 4.

The RBC locates initially at t=0 in an axial distance of $x_{0}$ from the inlet. The transverse location of the RBC is placed along the bisector of the first quadrant with a radial shift (r). Thus, the transverse coordinates of the RBC are $y_{0}=r$ and $z_{0}=r$, respectively as shown in Figure 2b (r is the radial shift). With this configuration, the RBC confinement is defined as the ratio between the effective RBC diameter $D_{r}=\sqrt{\frac{A_{0}^{tot}}{\pi }}$ and the domain height $L_{z}$:

(32) $$\chi =\frac{D_{r}}{L_{z}}.$$

The initial shape of the RBC is first set to be the idealized shape (Equation 1) for all simulation cases at the initial time t=0. A short period of relaxation $t_{\text{relax}}$ is allowed for the RBC under no external load (no flows) so that the internal forces of the RBC membrane balance. A uniform flow velocity U(t) is then applied at the channel inlet at $t>t_{relax}$ to induce the RBC’s deformation depending on the controlling strategy. The average shear rate across the channel height is defined as the ratio between the bulk velocity U(t) and the domain’s height:

(33) $$\bar{\overset{\cdot }{\gamma (t)}}=\frac{U(t)}{L_{z}}$$

#### 2.8.1 Constant shear rate condition $\left(I_{0}\right)$

Following the experimental study of Guckenberger et al (2018) (Channel-1, Table 4), FSI simulations of the RBC in channel flow with a constant flow rate are carried out with $x_{0}=22.5\;\mu m$. To highlight the constant flow rate, the notation $I_{0}$ is introduced to emphasize this condition. As shown in Table 5 , a constant inflow velocity $U(t)=\psi_{0}$ is required at the inlet of the computational domain. Two values of $\psi_{0}$ are considered: (*i*) $\psi_{0}=U_{3}=2\;mm/s$; and (*ii*) $\psi_{0}=U_{4}=6\;mm/s$. In these cases, two values of the radial shift are also investigated: $r_{1}=0$ and $r_{3}=0.7\;\mu m$. To simplify the discussions, the numerical values for the bulk velocity $\psi_{0}$ will not be explicitly referred to. Instead, only the acronyms $\left(U_{3}\right.$ and $\left.U_{4}\right)$ will be used for reasons that will be evident in the subsequent texts.

Using these notations, the FSI simulation cases are named using the convention for each type of inflow waveform (I), the bulk velocity (U), the radial shift (r), and the channel type, respectively. The first case $\left(I_{0}U_{3}r_{1}\chi_{1}\right)$ is configured with $\left(\psi_{0}=U_{3}=2\;mm/s\right)$ and $r=r_{1}=0\;\mu m$. The second case $\left(I_{0}U_{4}r_{3}\chi_{1}\right)$ is carried out with $\psi_{0}=U_{4}=6\;mm/s$ and $r=r_{3}=0.7\;\mu m$. Here, the Reynolds number is defined as $Re=\frac{UL_{s}}{\nu }$. The kinematic fluid viscosity of blood plasma is chosen as $\nu =\frac{\mu_{plasma}}{\rho }=1.2\times 10^{-6}{\;m}^{2}/s$. The summary of the parameters for each simulation case is shown in Table 5.

First, $t_{relax}=10\;ms$ and 7.0 *ms* are set for $I_{0}U_{3}r_{1}\chi_{1}$ (croissant) and $I_{0}U_{4}r_{3}\chi_{1}$ (slipper) simulations, respectively. After the relaxation period, a linear ramping period is set for each simulation case $t_{ramp}=30\;ms$ and 20 *ms* are set for $I_{0}U_{3}r_{1}\chi_{1}$ and $I_{0}U_{4}r_{3}\chi_{1}$, respectively. During this ramping period, the bulk velocity U(t) is linearly increased. The value of U(t) reaches $\psi_{0}$ at the end of the ramping period.

#### 2.8.2 Stepwise oscillatory flows ($I_{s}$)

**Fig. 3:**
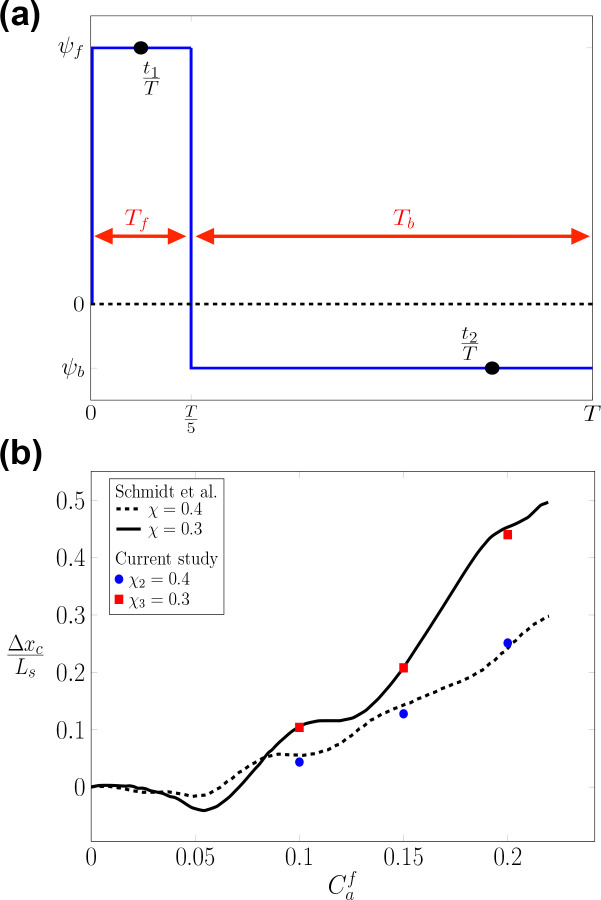
The propulsion step $\Delta x_{c}$ (Equation 36) as a function of the forward capillary number $Ca^{f}$ (Equation 35). The propulsion step $\Delta x_{c}$ is shown in terms of the length scale $\left(L_{s}=8\;\mu m\right)$. (*a*) The bulk flow waveform of the inflow (U(t)) has a stepwise shape (see Equation 34). Two-time instances ($\frac{t_{1}}{T}$ and $\frac{t_{2}}{T}$) are shown to exemplify the changes of RBC shapes over time. (*b*) Three values of $Ca^{f}=0.1,\;0.15$, and 0.2 (red squares and blue circles) are simulated. The computed values of $\Delta x_{c}$ are compared with Schmidt et al (2022) (solid lines).

To further validate our FSI model in oscillatory flows, the propulsion of the RBC in square channels is investigated (Schmidt et al, 2022). Two square channels (Channel-2 and Channel-3 in Table 4) with side lengths $L_{z}=16\;\mu m$ and 21 *μm* are used for the simulations, resulting in confinements $\chi_{2}=0.4$ and $\chi_{3}=0.3$, respectively. The initial location of the RBC is on the channel axis $\left(x_{0}=16\;\mu m,\;r=r_{1}=0\right)$. The computational configuration including the grid spacing, RBC surface meshes, and boundary conditions are shown in Figure 2a and Table 4. A stepwise asymmetric oscillatory waveform $I_{s}$ is used with two phases: (i) forward $T_{f}$; and (ii) backward $T_{b}$ periods $\left(\frac{T_{b}}{T_{f}}=4\right)$ as shown in Figure 3a. The velocities during the forward and backward phases are $\psi_{f}$ and $\psi_{b}\left(-\frac{\psi_{f}}{\psi_{b}}=4\right)$, respectively. The formula for the waveform is defined as:

(34) $$U(t)=\left\{\begin{array}{ll} \psi_{f} & \text{for}\;0\leq t<\frac{T}{5},\\ \psi_{b} & \text{for}\;\frac{T}{5}\leq t<T \end{array}\right.$$

Following this formula, the flow has a forward phase $\left(\psi_{f}>0\right)$ and a backward flow phase $\left(\psi_{b}<0\right)$. The maximum shear rate is defined as $\overset{-}{{\dot{\gamma }}_{f}}=\frac{\psi_{f}}{L_{z}}$.

The Capillary number $Ca^{f}$ in the forward flow phase is given as:

(35) $$Ca^{f}=\frac{4\psi_{f}L_{s}t_{R}}{L_{z}^{2}},$$

with $t_{R}=\frac{L_{s}\mu_{plasma}}{2\mu_{0}}$ and $\mu_{0}$ is the shear elastic modulus described in Table 2.

There are 6 values of $\psi_{f}$ are examined as $\psi_{1}=1.05$, $\psi_{2}=1.58$, $\psi_{3}=2.1$, $\psi_{4}=1.9$, $\psi_{5}=2.8$, and $\psi_{6}=3.7\;mm/s$, respectively. Following the naming convention of the simulations, six cases are formed with the respective parameters: $I_{s}\psi_{1}r_{1}\chi_{2}$; $I_{s}\psi_{2}r_{1}\chi_{2}$; $I_{s}\psi_{3}r_{1}\chi_{2}$; $I_{s}\psi_{4}r_{1}\chi_{3}$, $I_{s}\psi_{5}r_{1}\chi_{3}$, $I_{s}\psi_{6}r_{1}\chi_{3}$ as shown in Table 5. As the waveform applied is of a stepwise nature, there is no relaxation time taken into consideration for these cases $\left(t_{relax}=0\right)$.

Under these oscillatory conditions, the axial propulsion step $\left(\Delta x_{c}\right)$ is recorded at the end of the forward time interval of the asymmetric oscillating flow $\left(t=T_{f}=\frac{T}{5}\right)$, as a function of the forward (peak) capillary number $Ca^{f}$ for the chosen shear rates (Schmidt et al, 2022). Thus $\Delta x_{c}$ is defined as the displacement of the RBC’s centroid (C) at the end of the forward phase $\left(t=\frac{T}{5}\right)$:

(36) $$\Delta x_{c}=x_{c}(t=\frac{T}{5})-x_{c}(t=0)$$

#### 2.8.3 Sinusoidal flow simulations

To study the effect of the pulsatile flow on the propulsion and the behaviour of the cellular response (morphology changes) of the RBC, we considered time-periodic flow U(t). The flow time period consists of three separate phases: (i) the forward $\left(T_{f}\right)$; (*ii*) the resting $\left(T_{r}\right)$, and the backward $\left(T_{b}\right)$ periods. The oscillatory frequency is chosen to be f=20Hz (Mutlu et al, 2018) and thus $=T_{f}+T_{r}+T_{b}=50\;ms$. The asymmetry of the waveform is adjusted by changing the values of $T_{f}$, $T_{r}$, and $T_{b}$. The formula for the waveform is:

(37) $$U(t)=\left\{\begin{array}{ll} A\;sin(2\pi \frac{t}{T_{f}}) & \text{for}\;0\leq t\leq T_{f},\\ 0 & \text{for}\;T_{f}\leq t\leq T_{f}+T_{r}\\ A\;sin(2\pi \frac{\left(t-T_{f}-T_{r}\right)}{T_{b}}) & \text{for}\;T_{f}+T_{r}\leq t\leq T \end{array}\right.$$

**Fig. 4:**
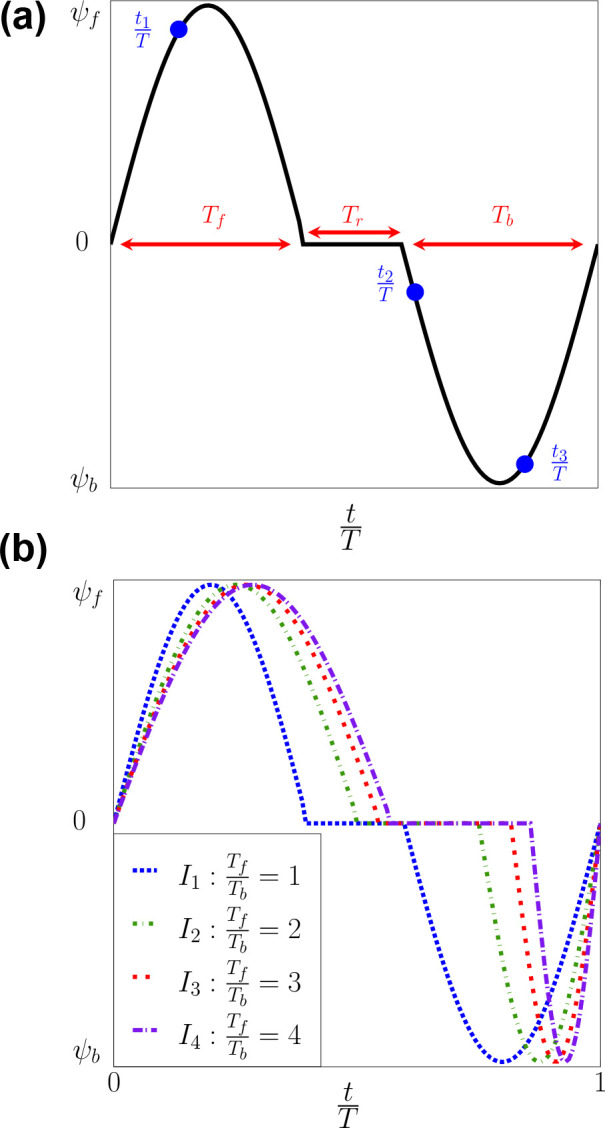
(*a*) Sinusoidal inflow velocity U(t) profile with the forward $\left(T_{f}\right)$, resting $\left(T_{r}\right)$ and backward $\left(T_{b}\right)$ time intervals. (*b*) Four inflow types with different $\frac{T_{f}}{T_{b}}$ rations are considered (see also Table 6). The time instances $\frac{t_{1}}{T}$, $\frac{t_{2}}{T}$ and $\frac{t_{3}}{T}$ shown in (*a*) represent instances at which the RBC shapes are recorded. The exact values of the time instances are shown in Figure 8 and Tables 9–12.

The symmetric waveform $\left(I_{1}\right)$ is created with $T_{f}=T_{b}$ (completely symmetric). The asymmetric waveforms ($I_{2}$, $I_{3}$, and $I_{4}$) are formed by progressively reducing the period of $T_{b}$. Four distinct inflow types were generated with symmetry and asymmetric waveforms ($I_{1}$, $I_{2}$, $I_{3}$, and $I_{4}$) as seen in Figure 4 and Tables 6 and 7. For each of these waveforms, three different velocity magnitudes $\left(A=U_{1},U_{2}\right.$ and $\left.U_{3}\right)$ and three different radial shifts ($r_{1}$, $r_{2}$ and $r_{3}$) are considered as shown in Tables 8. In total, the combinatoric arrangements lead to a total of 36 distinct simulation cases with the notation $I_{m}U_{n}r_{p}\chi_{1}$ with the corresponding values of the indices =1,2,3,4, n=1,2,3, and p=1,2,3. The outline of the simulation cases is shown in Table 8. In addition, the RBC shapes are recorded over a time period of two cycles 2T as exemplified in Figure 4b, in which the initial location of the RBC is set at $x_{0}=22.5\;\mu m$. Due to the nature of the sinusoidal waveform applied, there was no relaxation time for all of these cases $\left(t_{relax}=0\right)$. The centroid’s displacement is monitored continuously as the function of time:

(38) $$\Delta x_{c}(t)=x_{c}(t)-x_{c}(t=0)$$

## 3 Results

### 3.1 Model validation

#### 3.1.1 Coarse-graining validation

To first validate the coarse-graining procedure employed in our study, a stretching test is carried out and aimed to replicate the experimental test of Mills et al (2004). In our simulations, the parameters to describe the physical characteristics of the RBC are listed in Table 2. Following the coarse-graining procedure, the model parameters for the cell membrane such as the equilibrium length, the persistence length, the spring stiffness, and the spontaneous angle are computed for each value of $N_{v}$ as in Table 3. The cytosol fluid is modeled by a set of particles $N_{f}=100$, placed within the interior volume of the cell membrane as shown in Figure 1b.

In this experiment, two external forces $F_{stretch}$ and $-F_{stretch}$ with opposite directions are applied on both sides of the RBC. The magnitude of the force $F_{stretch}$ is increased in a stepwise manner from 0 to 200pN (a total of 16 steps). The axial diameter $\left(D_{a}\right)$ and transverse diameter $\left(D_{t}\right)$ are measured for every step. The definitions of $D_{a}$ and $D_{t}$ are shown in Figure 1a. The simulations are performed systematically with different RBC surface mesh resolutions by changing the number of vertices $\left(N_{v}\right)$.

The current RBC model accurately replicates the elastic response of the RBC under stretching forces, as revealed by the results of $D_{a}$ and $D_{t}$ in Figure 1. During stretching, the dynamic response of cytosol particles is visible indicating the coupling between the membrane and the cytosol fluid. The shapes of the RBC under loading conditions agree with ones from experimental data of Mills et al (2004). The computed values of the axial $\left(D_{a}\right)$ and transverse $\left(D_{t}\right)$ diameters agree well with the experimental values as seen in Figure 1a. In particular, the values of $D_{a}$ and $D_{t}$ are consistent across the different values of $N_{v}$, which indicates a robust performance of the coarse-graining procedure. There is a disagreement between the simulated results and the experimental value of $D_{t}$. Examining the shapes of the RBC in the simulations (Figure 1b), it is revealed that the RBC tends to rotate around the stretching direction. This rotation leads to the difference between the experimental and numerical results of $D_{t}$. In brief, the mechanics of RBC is well replicated by the computational model across different level of coarse-graining. The value of $N_{v}=1000$ is chosen to report the dynamics of the RBC in subsequent sections.

#### 3.1.2 Deformation of the RBC under a constant shear rates $\overset{-}{\dot{\gamma_{0}}}$

**Fig. 5:**
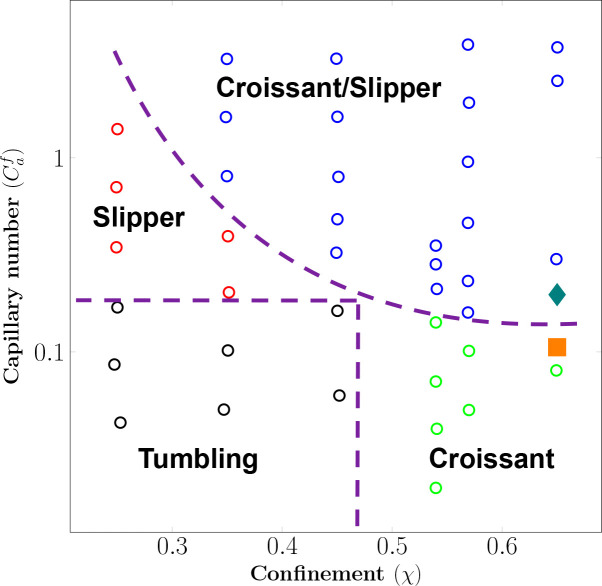
Validation with the shape diagram (Ca,χ) of Agarwal and Biros (2022) (unfilled circles ) for flows of RBC in a confined channel. The dash lines depict distinct regions representing different dynamics/shapes of RBC. Our simulations for the croissant shape ($I_{0}U_{3}r_{1}\chi_{1}$ - filled square) and the slipper shape ($I_{0}U_{4}r_{1}\chi_{1}$ - filled diamond) agree well with the reported regions with $\chi =\chi_{1}=0.65$.

**Fig. 6:**
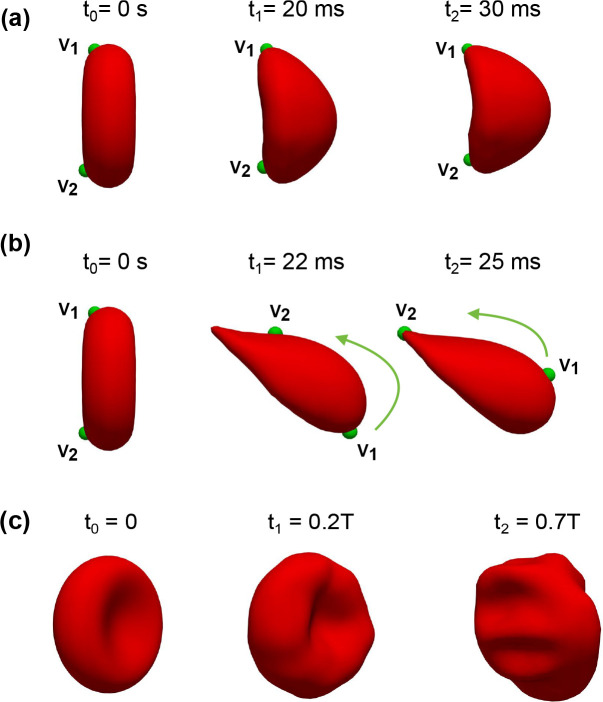
The transitions from the idealized shape to realistic shapes under the impact of shear flows. The stable shapes are attained under the impact of a constant shear rate $\left(I_{0}\right)$ in: (*a*) croissant shape $\left(I_{0}U_{3}r_{1}\chi_{1}\right)$ and (*b*) slipper shape $\left(I_{0}U_{4}r_{3}\chi_{1}\right)$. The RBC membrane only exhibits the tank-treading effect in the slipper shape $\left(I_{0}U_{4}r_{3}\chi_{1}\right)$, which is characterized by the motions of two Lagrangian markers $V_{1}$ and $V_{2}$. The slipper shape is maintained by the counter-clockwise rotation (the green arrow) of the cellular membrane around the RBC’s centroid. The multilobe shape appears (*c*) under the oscillatory flow $\left(I_{s}\psi_{6}r_{1}\chi_{3}\right)$ during the backward phase (0.7T).

Under constant shear rate conditions $\left(I_{0}\right)$ as described in section 2.8.1, two districts of the RBC shape are observed: (*i*) the croissant shape $\left(I_{0}U_{3}r_{1}\chi_{1}-\overset{-}{\dot{\gamma_{0}}}=200\;s^{-1}\right)$; and (*ii*) the slipper shape $\left(I_{0}U_{4}r_{3}\chi_{1}-\overset{-}{\dot{\gamma_{0}}}=600{\;s}^{-1}\right)$ as shown in Figure 5 and Figure 6.

Under low shear rate $\left(I_{0}U_{3}r_{1}\chi_{1}\right)$, the RBC is initially placed along the centerline of the microchannel (discocyte shape). As the RBC interacts with the incoming flow, deforms, and eventually transitions to a croissant shape as shown in Figure 6a. The terminal shape (croissant) is attained as the RBC continues to propagate along the channel’s symmetry axis. Note that the croissant shape, in this case, is not fully axis-symmetric.

Under high shear rate $\left(I_{0}U_{4}r_{3}\chi_{1}\right)$, the RBC transitions to the slipper shape as shown in Figure 6b, which exhibits a bistability mode with tank-treading behaviour. Note that the RBC is placed at a radial shift $r_{3}=0.7\;\mu m$. Thus the initial location of the RBC is not at the channel’s symmetry axis. The tank-treading effect is a complex dynamic in which the RBC membrane propagates axially along the channel while it rotates around its own center of mass as seen in Figure 6b. A counter-clockwise rotation is observed as indicated by the locations of two membrane particles (Lagrangian points - $V_{1}$ and $\left.V_{2}\right)$ at different time instances $\left(t_{1}=22\;ms\right.$ and $\left.t_{2}=25\;ms\right)$.

In both the croissant or slipper shapes, the transition from the initial shape (discocyte) to the terminal shape (either croissant or slipper) occurs within around 30 *ms* as seen in Figure 6. These transitions agree well with the corresponding experimental data of Guckenberger et al (2018) as well as described in recent experiments on RBC transient dynamics (Recktenwald et al, 2022; Prado et al, 2015). Furthermore, these transitions are in good agreement with the shape diagram produced by Agarwal and Biros (2022) for different Capillary numbers and confinements as seen in Figure 5. In conclusion, our simulations are able to replicate the dynamics of the croissant and slipper shapes excellently.

**Fig. 7:**
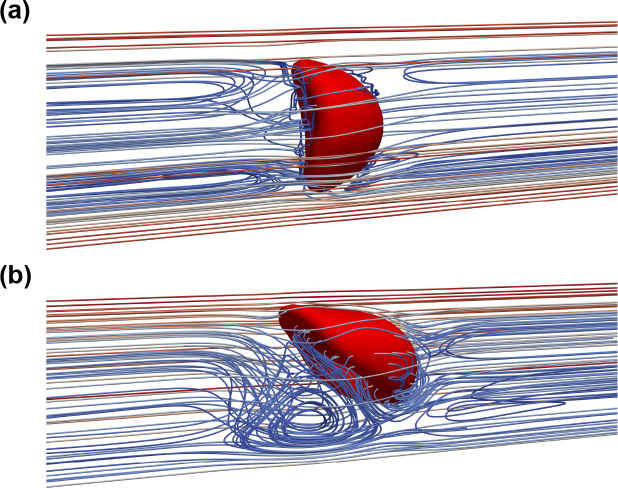
The extracellular flow patterns for: (*a*) the croissant shape $\left(I_{0}U_{3}r_{1}\chi_{1}\right)$ and (*b*) the slipper shape $\left(I_{0}U_{4}r_{3}\chi_{1}\right)$. The flow streamlines are reconstructed using the co-moving frame method as discussed in section 3.1.2. The tank-treading effect induces a closed vortex to form on the upstream side of the RBC.

The extracellular patterns of the croissant and slipper shapes also agree well with the experimental data of Guckenberger et al (2018). The extracellular flow pattern can be visualized by reconstructing the relative flow velocity field (Yaya et al, 2021) (co-moving frame). The relative velocity is defined as the difference between the flow velocity and the RBC’s centroid velocity as shown in Figure 7. In the croissant shape $\left(I_{0}U_{3}r_{1}\chi_{1}\right)$, the velocity streamlines closely resemble an axi-symmetrical flow pattern (Figure 7a). The downstream side of the RBC membrane deforms significantly whereas the upstream side barely changes as depicted in Figure 7b. In the slipper shape $\left(I_{0}U_{4}r_{3}\chi_{1}\right)$, there exists an asymmetrical vortical structure in the vicinity of the RBC membrane. As the slipper shape emerges, a fully closed vortex ring is created by a separated flow region, which is close to the channel wall. In short, the emergence of the RBC shape dictates the extracellular flow pattern.

#### 3.1.3 Propulsion of RBC under stepwise oscillatory flows $\left(I_{s}\right)$

Under stepwise flow waveform $\left(I_{s}\right)$, our simulation results agree well with the propulsion step map $\left(\Delta x_{c},\;Ca^{f}\right)$, which was developed by Schmidt et al (2022) for both channels $\chi_{2}=0.5$ and $\chi_{3}=0.38$. In both cases, the propulsion step $\left(\Delta x_{c}\right)$ is observed to monotonically increase with the values of $Ca^{f}$. However, the $\Delta x_{c}$ is higher in the lower confinement channel $\left(\chi_{3}\right)$, which indicates the importance of channel confinement.

In all simulation cases ($I_{s}\psi_{1}r_{1}\chi_{3}$, $I_{s}\psi_{2}r_{1}\chi_{3}$, $I_{s}\psi_{3}r_{1}\chi_{3}$, $I_{s}\psi_{4}r_{1}\chi_{3}$, $I_{s}\psi_{5}r_{1}\chi_{3}$, and $I_{s}\psi_{6}r_{1}\chi_{3}$), the RBC transitions from the idealized discocyte to the biconcave shape during the forward phase $\left(0<t<\frac{T}{5}\right)$ with all values of the peak forward flow $\left(\psi_{f}=1.05\;mm/s\right.$ to $\left.\psi_{f}=4.34\;mm/s\right)$ as shown in Figure 6c. Strikingly, the complex multilobe shape emerges during the backward phase $T_{b}$. The elastic response of the RBC membrane to the oscillatory flow during the cycle T is depicted for the case $I_{s}\psi_{6}r_{1}\chi_{3}$ in Figure 6c. The reversal of the flow direction during $T_{b}$ results in membrane buckling and stretching, which give rise to the multilobe shape. Note that these cases are placed initially at the channel center $\left(r=r_{1}=0\right)$. Therefore, it is possible to induce complex dynamics of RBCs in a confined channel by changing the inflow waveform only.

### 3.2 The impact of sinusoidal flows on RBC dynamics

#### 3.2.1 The emergence of RBC shapes

**Fig. 8:**
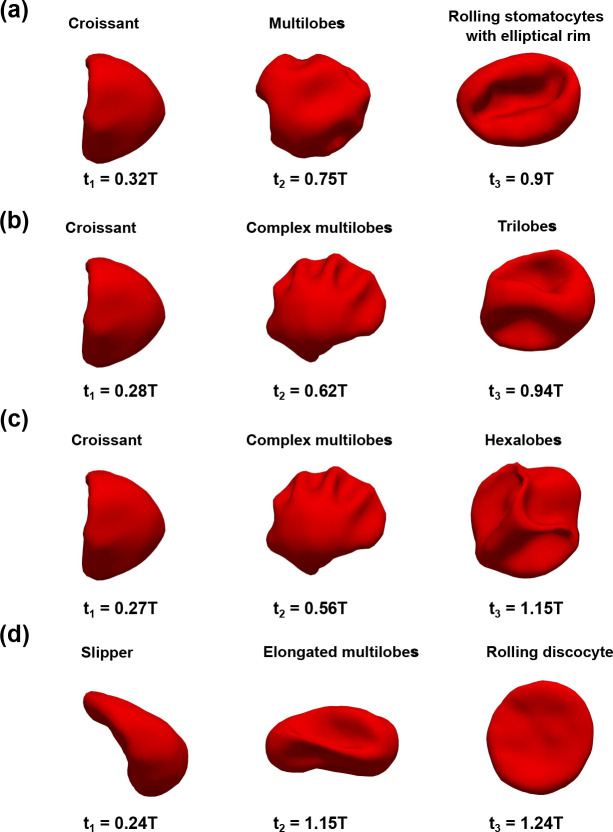
The emergence of complex shapes induced by different inlet sinusoidal waveforms at different time instances in the flow cycle $t_{1}$ (end of forward phase), $t_{2}$ (during the resting period) and $t_{3}$ (backward flow phase). (*a*) $I_{1}U_{1}r_{1}\chi_{1},$ (*b*) $I_{3}U_{2}r_{1}\chi_{1}$, (*c*) $I_{4}U_{2}r_{1}\chi_{1}$ and (*d*) $I_{4}U_{3}r_{3}\chi_{1}$.

The sinusoidal flow waveform ($I_{1}$, $I_{2}$, $I_{3}$, and $\left.I_{4}\right)$ further adds complexity to the membrane dynamics as the shape of RBC is highly sensitive to the extracellular flow condition. As a result of the pulsatile flow condition, the RBC shape continuously responds to the applied flow in the channel. Our simulations show that the RBC alternates its shapes in one of the following types: 1) croissant; 2) slipper; 3) trilobes; 4) simple/complex/elongated multilobes; 5) rolling stomatocytes; 6) hexalobes; and 7) rolling discocyte as shown in Figure 8 and Tables 9–12. The emergence of each type will be discussed as follows.

In all cases, the RBC evolves to the croissant (C) or the slipper (S) shape during the forward phase $\left(0<t<T_{f}\right)$ of the flow cycle $\left(\frac{t}{T}\approx 0.25\right)$ as shown in Tables 9–12. This process is demonstrated for the cases $I_{1}U_{1}r_{1}\chi_{1}$, $I_{3}U_{2}r_{1}\chi_{1}$, $I_{4}U_{2}r_{1}\chi_{1}$, and $I_{4}U_{3}r_{3}\chi_{1}$ as seen in Figure 8 (first column). Note that the transition to C or S mode from the biconcave shape is dependent on the value of the radial shift (r). As shown in Tables 9–12, the S mode appears only when the RBC is initially placed not exactly at the cross-sectional center (r>0) (for example, $I_{4}U_{3}r_{3}\chi_{1}$). The RBC remains in C mode during the forward phase if it is initially placed at the cross-section center (r=0) regardless of the bulk flow waveform either $I_{1}$, $I_{2}$, $I_{3}$, or $I_{4}$ as seen in cases $I_{1}U_{1}r_{1}\chi_{1}$, $I_{3}U_{2}r_{1}\chi_{1}$, and $I_{4}U_{2}r_{1}\chi_{1}$. In brief, the croissant and the slipper shapes exist during the forward phase and their emergence depends on the radial shift (r).

The RBC transitions from the simple shapes (croissant and slipper) toward more complex shapes (trilobes, simple/complex/elongated multilobes, rolling stomatocytes, hexalobes, and rolling discocyte) later in the flow cycle during the resting/reverse periods $\left(\frac{t}{T}>0.5\right)$ as shown in Tables 9–12 and Figure 8 (middle column). The shape transformation is initiated by the buckling of the RBC membrane, which takes place in the resting interval $\left(T_{r}\right)$ (see Figure 4) of the flow. As a result of the change in flow direction in $T_{b}$, the RBC experiences considerable stretching and compression, leading to significant alterations in its membrane shape as seen in Figure 8. Note that the RBC does not return to either C or S mode at the beginning of the second cycle T<t<2T as shown in Tables 9–12. Thus the shape transition process is irreversible even if the inflow waveform is symmetric $\left(I_{1}\right)$ as shown in Table 9.

#### 3.2.2 The impacts of the initial position (the radial shift - r)

Our results in Figure 8 show that the initial position (r) plays a critical role in the emergence of RBC shapes. The RBC is placed initially at the channel axis $r=r_{1}=0$ under both the symmetric $\left(I_{1}U_{1}r_{1}\chi_{1}\right)$ and asymmetric ($I_{3}U_{2}r_{1}\chi_{1}$ and $I_{4}U_{2}r_{1}\chi_{1}$) waveforms in Figure 8a, Figure 8b, and Figure 8c, respectively. In these cases $\left(I_{1}U_{1}r_{1}\chi_{1}\right.$, $I_{3}U_{2}r_{1}\chi_{1}$, and $I_{4}U_{2}r_{1}\chi_{1}$), the RBCs all transition sequentially from the croissant shape toward the multilobes/complex multilobes, and finally one of the poly-lobes (trilobes, rolling stomatocytes, or hexalobes). When $r=r_{3}>0$ in the case $I_{4}U_{3}r_{3}\chi_{1}$, the RBC remains mostly the slipper shape during the forward phase $\left(t<T_{f}\right)$ as seen in Figure 8d. It transitions toward the elongated multilobe during the backflow phase $\left(T_{f}+T_{r}<t<T\right)$. Finally, the RBC becomes a rolling discocyte in the second cycle (t≈1.2T). Note that $r_{3}$ is a small value (0.7μm) and thus the shape transition process is strongly sensitive to the initial placement of the RBC. The dependence of RBC dynamics on the value of r is explained below.

**Fig. 9:**
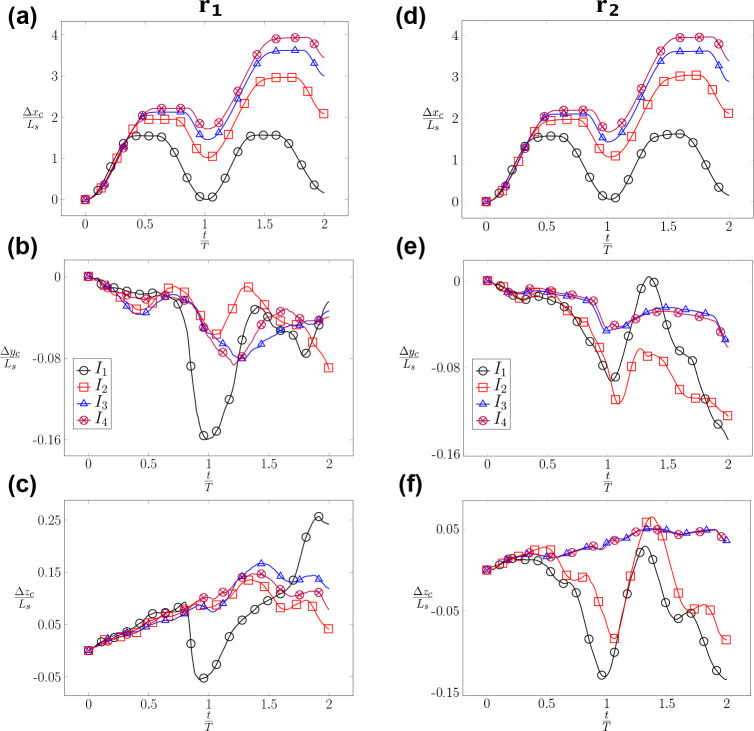
The impacts of the radial shift r on the time evolution of the RBC’s centroid displacement $\left(\Delta x_{c},\;\Delta y_{c},\;\Delta z_{c}\right)$. The instantaneous evolution of the RBC’s centroid position $\left(x_{c},y_{c},z_{c}\right)(t)$ is recorded as the RBC propagates along the channel. The displacements of the RBC from its initial location along three directions $\left(\Delta x_{c},\;\Delta y_{c},\;\Delta z_{c}\right)$ are measured in units of the length scale $L_{s}$. The evolution of the centroid position is examined under two conditions: (*i*) centred initial position $r=r_{1}=0$ (left column- (a-c) for the cases $I_{1}U_{1}r_{1}\chi_{1}$, $I_{2}U_{1}r_{1}\chi_{1}$, $I_{3}U_{1}r_{1}\chi_{1}$, and $I_{4}U_{1}r_{1}\chi_{1}$; and (*ii*) off-centered initial position $r=r_{2}=0.4\;\mu m$ (right column - (d-f) for the cases $I_{1}U_{2}r_{2}\chi_{1}$, $I_{2}U_{2}r_{2}\chi_{1}$, $I_{3}U_{2}r_{2}\chi_{1}$, and $I_{4}U_{2}r_{2}\chi_{1}$ as described in Table 7.

When the RBC is positioned at the center line (r=0) of the channel, its migration is highly dependent on the type of inflow waveform. When the RBC is subjected to a symmetric waveform $\left(I_{1}\right)$ as shown in Figure 9a, it oscillates around its initial position with a minimal migration along the channel (axial) direction. However, as the inflow profile transitions to an asymmetric waveform ($I_{2}$, $I_{3}$, and $\left.I_{4}\right)$ with an increasing $T_{f}$, the RBC gains more momentum during the forward phase and propels far away from its initial position as shown in Figure 9a (left). The value of the axial displacement $\left(\Delta x_{c}\right)$ (t) increases sequentially from $I_{1}$, $I_{2}$, $I_{3}$ to $I_{4}$. $\Delta x_{c}$ reaches a maximum value of approximately $4\times L_{s}$ at the end of the second cycle for the case $I_{4}$. In the lateral direction, $\Delta y_{c}$ reaches a value of approximately $0.16\times L_{s}$ at the end of the first cycle for the symmetric waveform $\left(I_{1}\right)$ as shown in Figure 9b (left). For the cases $I_{2}$, $I_{3}$, and $I_{4}$ the values of $\Delta y_{c}$ remains comparably low $\left(\approx 0.08\times L_{s}\right)$ during the cycles. Furthermore, the vertical displacement $\Delta z_{c}$ as seen in Figure 9c follows a monotonically upward trend throughout the second cycle. This results in a maximum vertical displacement $\Delta z_{c}$ of approximately $0.25\times L_{s}$ for $I_{1}$. For other waveforms ($I_{2}$, $I_{3}$, and $I_{4}$), a smaller upward trend is observed with a vertical displacement $\Delta z_{c}\approx 0.08\times L_{s}$ at the end of the first cycle. During the second cycle, the cell is observed to migrate downward. In summary, the symmetric waveform $\left(I_{1}\right)$ leads to a maximum displacement in the transverse directions while the asymmetric waveforms ($I_{2}$, $I_{3}$, and $I_{4}$) result in the maximum axial displacement for r=0.

As the RBC is placed at the off-centered location $\left(r=r_{2}=0.4\;\mu m\right)$ as seen in Figure 9d, its axial migration is similar to ones with r=0 (Figure 9a). The axial displacement $\left(\Delta x_{c}\right)$ is dependent on the applied inflow waveform and also reaches $4\times L_{s}$ for the $I_{4}$ waveform. These values indicate that the radial shift (r) does not significantly affect the axial migration of the RBC. However, the lateral migration of RBC in Figure 9e and Figure 9f does show a dependence of the applied waveform. The values of $\Delta y_{c}$ and $\Delta z_{c}$ reach $0.14\times L_{s}$ for $I_{1}$ and $I_{2}$ at the end of the second cycle. Surprisingly, the impact of the applied waveform seems to diminish as the value of $T_{f}$ increases. Comparing the case $I_{3}U_{2}r_{2}\chi_{1}$ and $I_{4}U_{2}r_{2}\chi_{1}$ ($I_{3}$ and $I_{4}$), the values of $\Delta y_{c}$ and $\Delta z_{c}$ in Figure 9e and Figure 9f are nearly identical, resulting in a vertical displacement of approximately $0.04\times L_{s}$. To summarize, the impact of the applied waveform is significant on the axial displacement of the RBC but it is milder on the lateral displacements when r>0. The impact of the applied waveform diminishes as the forward time $T_{f}$ increases.

#### 3.2.3 The impacts of the waveform (I)

**Fig. 10:**
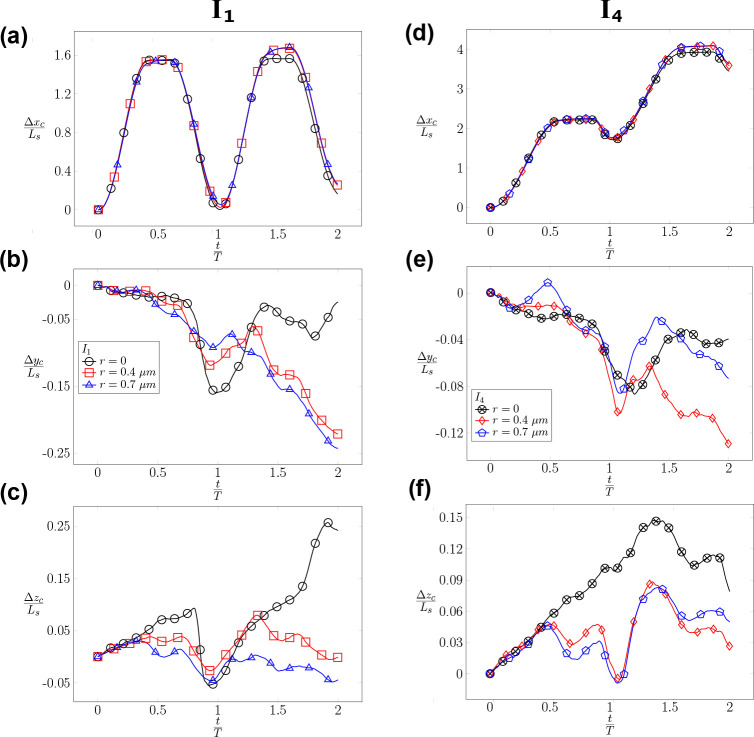
The impacts of the waveform (I) on the time evolution of the RBC’s centroid displacement $\left(\Delta x_{c},\;\Delta y_{c},\;\Delta z_{c}\right)$. The instantaneous evolution of the RBC’s centroid position $\left(x_{c},y_{c},z_{c}\right)(t)$ is recorded as the RBC propagates along the channel. The displacements of the RBC from its initial location along three directions $\left(\Delta x_{c},\Delta y_{c},\Delta z_{c}\right)$ are measured in units of the length scale $L_{s}$. The evolution of the centroid position is examined under two conditions: (i) the symmetric $I_{1}$ (left column- (a-c)); and (ii) the asymmetric $I_{4}$ (right column - (d-f)) waveforms at different values of the radial shift $r_{1}$, $r_{2}$, and $r_{3}$. The symmetric flow cases (left column) include $I_{1}U_{1}r_{1}\chi_{1}$, $I_{1}U_{1}r_{2}\chi_{1}$, and $I_{1}U_{1}r_{3}\chi_{1}$. The asymmetric flow cases (right column) include $I_{4}U_{1}r_{1}\chi_{1}$, $I_{4}U_{1}r_{2}\chi_{1}$, and $I_{4}U_{1}r_{3}\chi_{1}$ cases. The exact values of $r_{1}$, $r_{2}$, and $r_{3}$ are described in Table 7.

It is striking to observe the irreversible dynamics of RBC in Figure 10. When the RBC is subjected to the symmetric waveform $\left(I_{1}\right)$ with different radial shifts $r=r_{1},r_{2}$ and $r_{3}$, the RBC oscillates around its initial position with a minimal displacement along the axial direction as shown in the cases $I_{1}U_{1}r_{1}\chi_{1}$, $I_{1}U_{1}r_{2}\chi_{1}$, and $I_{1}U_{1}r_{3}\chi_{1}$. Despite the inflow waveform being completely symmetrical (a sine function - $I_{1}$), the axial position of the RBC in Figure 10a (left column) shows a net positive value of the displacement $\Delta x_{c}$ at the end of the first (t=T) and second cycle (t=2T) even when there is no radial shift (r=0). Though small, this positive value of $\Delta x_{c}$ indicates that the RBC does not go back exactly to its initial location, which is $\Delta x_{c}(t=0)=0$. At all values of the radial shift of r=0,0.4, and 0.7 *μm*, this irreversible dynamics is even more evident as shown in the lateral displacements in Figures 10b–c. The magnitudes of $\Delta y_{c}$ and $\Delta z_{c}$ are comparable for all values of r during the cycles. For the case $I_{1}U_{1}r_{1}\chi_{1}$ (r=0) the value of $\Delta y_{c}$ reaches a value of approximately $0.16\times L_{s}$ at the end of the first cycle. For the cases $I_{1}U_{1}r_{2}\chi_{1}$ and $I_{1}U_{1}r_{3}\chi_{1}$, the values of $\Delta y_{c}$ and $\Delta z_{c}$ reach approximately $0.25\times L_{s}$ at the end of the second cycle. In the vertical direction $\left(z_{c}\right)$ in Figure 10c, the well-centered RBC(r=0) is influenced by the change of flow direction, which is depicted by the upward and downward trends in the first cycle. However, the cell follows a dominant upward trend during the entire second cycle resulting in a lateral migration of around $0.25\times L_{s}$. Therefore, there exists a significant lateral migration of the RBC during its propagation regardless of its initial position under the symmetrical waveform condition $\left(I_{1}\right)$. In conclusion, a symmetrical flow waveform $\left(I_{1}\right)$ results in minimal propulsion along the axial direction but a significant lateral migration.

Under asymmetric waveform $I_{4}$, the RBC propels along the channel direction with an axial displacement of approximately $2\times L_{s}$ in each cycle as shown in Figure 10d. As the waveform becomes asymmetric with a longer forward phase, the RBC does not go back significantly during the reverse phase. It rather remains at a displacement value of $\Delta x_{c}\approx 1.9\times L_{s}$ at the end of the first cycle. It continues to propel in the second cycle up to $\Delta x_{c}\approx 4.0\times L_{s}$. Surprisingly, the lateral displacements of the RBC $\left(\Delta y_{c},\Delta z_{c}\right)$ are smaller in comparison to ones in the symmetric case $\left(I_{1}\right)$. The values of $\left(\Delta y_{c},\Delta z_{c}\right)$ are within $0.15\times L_{s}$ for all cases $I_{4}U_{1}r_{1}\chi_{1}$, $I_{4}U_{1}r_{2}\chi_{1}$, and $I_{4}U_{1}r_{3}\chi_{1}$ as shown in Figure 10e and f. In brief, the RBC propels significantly under the impact of the asymmetrical flow waveform $I_{4}$ along the axial direction but it does not migrate significantly in the lateral directions.

#### 3.2.4 Extracellular flow dynamics at the vicinity of the RBC under oscillatory flows

**Fig. 11:**
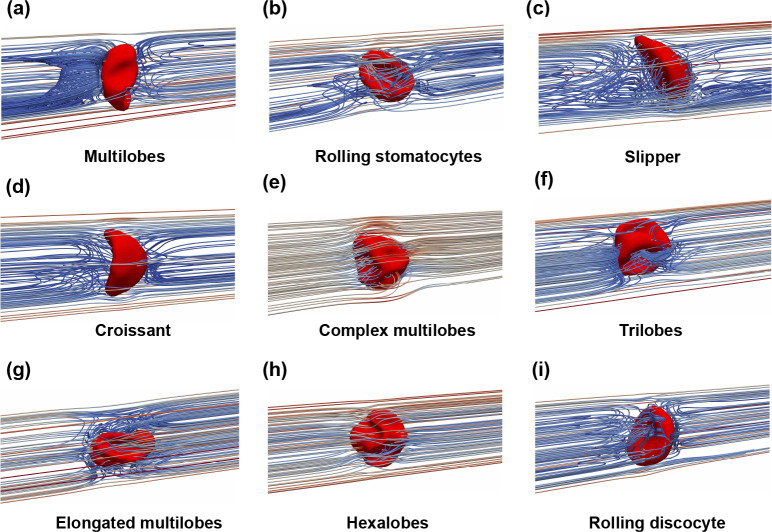
Flow patterns surrounding the RBC under different membrane shapes (see also Figure 8). The flow patterns are visualized using streamlines of the velocity field in the co-moving frame with the RBC (see also section 3.1.2). The representative flow patterns are shown for: (a) the multilobes $\left(I_{1}U_{1}r_{1}\chi_{1}\right)$; (b) rolling stomatocytes $\left(I_{1}U_{1}r_{1}\chi_{1}\right)$; (c) slipper $\left(I_{1}U_{3}r_{3}\chi_{1}\right)$; and (d) croissant $\left(I_{1}U_{3}r_{1}\chi_{1}\right)$; (e) complex multilobes $\left(I_{1}U_{3}r_{1}\chi_{1}\right)$; (f) trilobes $\left(I_{1}U_{3}r_{1}\chi_{1}\right)$; (g) elongated multilobes $\left(I_{1}U_{3}r_{1}\chi_{1}\right)$; (h) hexalobes $\left(I_{4}U_{2}r_{1}\chi_{1}\right)$; and (i) rolling discocyte $\left(I_{1}U_{1}r_{2}\chi_{1}\right)$.

The emergence of the RBC shape has a close relationship with the flow pattern of the surrounding fluid (extracellular flow). Under the impact of the channel confinement, the deformation of RBC is well regulated by the flow waveform, which results in distinct extracellular flow patterns surrounding the RBC as shown in Figures 8 and Figure 11. To highlight the impact of the RBC motion, the flow pattern is visualized in the co-moving frame with the RBC’s centroid (see section 3.1.2). Thus, the flow streamlines are represented from the perspective of the RBC.

The case $I_{1}U_{1}r_{1}\chi_{1}$ is selected to illustrate the evolution of flow pattern as the RBC deforms from a relatively simple shape to a more complicated shape as depicted in Figure 11a and Figure 11b. This case is chosen because the temporal variation of the waveform is completely symmetrical $\left(I_{1}\right)$. Moreover, the RBC is placed initially at the channel axis $\left(r=r_{1}=0\right)$ with the lowest forward velocity $\psi_{f}=U_{1}=1\;mm/s$. In the case $I_{1}U_{1}r_{1}\chi_{1}$, Figure 11a revealed that the RBC has a multilobes shape at the end of the forward phase. The presence of the large lobes results in a more convoluted streamlines pattern during the resting phase. As the RBC undergoes a morphological transition to rolling stomatocytes at the end of the first cycle (t=0.9T), the streamlines exhibit changes (Figure 11b). During the second cycle, the RBC gradually transforms into a rolling discocyte by the end of the second cycle (t=1.8T).

The impact of the radial shift (r) on the RBC shape and the resulting flow pattern is significant. To highlight the impact of the initial location, the case $I_{1}U_{3}r_{3}\chi_{1}$ was selected to visualize the flow patterns. As shown in Figure 11c, due to the off-centered initial location (r>0) the slipper shape emerges during the forward phase. A closed vortex ring is also observed downstream of the RBC as the flow velocity reaches its maximum magnitude in the forward phase. This phenomenon is similar to the one observed in the constant shear rate case ($I_{0}U_{4}r_{3}\chi_{1}$ with $\left.U_{4}=6\;mm/s\right)$ in Figure 7b. This is remarkable since the peak flow $\psi_{f}$ is rather three times lower in this case $\psi_{f}=U_{3}=2\;mm/s$.

The case $I_{1}U_{3}r_{1}\chi_{1}$ (Figures 11d, 11e, 11f, and 11g) is selected to illustrate further the impact of the peak forward flow $\psi_{f}$. In this case, the peak velocity is $\psi_{f}=U_{3}=2\;mm/s$. In comparison to the case $I_{1}U_{1}r_{1}\chi_{1}$ (Figure 11a and Figure 11b), only the value of the peak velocity $\psi_{f}$ is increased. However, the RBC shape evolves in a completely different sequence as opposed to the case $I_{1}U_{1}r_{1}\chi_{1}$. During the forward phase, the RBC transitions quickly to the croissant shape in Figure 11d at (t=0.28T), just after the peak forward flow. The flow patterns are similar to those observed under case $I_{0}U_{3}r_{1}\chi_{1}$ in Figure 7a. During the resting period $\left(T_{f}<T<T_{f}+T_{r}\right)$, the flow velocity surrounding the cell decreased notably and the complex multilobes shape emerges as seen in Figure 11e. The flow pattern is perturbed minimally surrounding the RBC as its shape turns to the trilobes shape as depicted in Figure 11f. During the forward flow phase of the second cycle, the elongated multilobes appear (t=1.2T).

The overall dynamics of the RBC over the cycles depend significantly on the radial shift. As demonstrated in Table 9 to Table 12, the multilobes shape appears at the beginning of each cycle if the RBC is located initially at the channel center r=0. Under the specific condition of the case $I_{4}U_{2}r_{1}\chi_{1}$, the multilobes shape can further transform into the hexalobes shape during the forward flow phase of the second cycle (t=1.15T) as shown in Table 12. Its corresponding flow patterns are shown in Figure 11h, in which the extracellular flow was observed to exhibit a minimal disturbance around the hexalobes shape as the RBC. For the majority of the off-centered cases (r>0), the rolling discocyte is the most commonly found as shown in Table 9 to Table 12. The flow pattern around a discocyte is exemplified in Figure 11i for the case $I_{1}U_{1}r_{2}\chi_{1}$ at t=1.25T. Here, the flow streamlines show a distinct separation of upstream and downstream regions.

## 4 Discussion

Due to the membrane flexibility, RBC deforms swiftly under the shear flow(Lanotte et al, 2016). This morphological feature can be exploited to understand the mechanical properties of the RBC membrane (Prado et al, 2015) and thus it has the potential to identify the pathological changes (Recktenwald et al, 2022) of RBC’s membrane. However, the exact mechanism of this response is not yet fully understood. In this work, we explore the impacts of the unsteady shear rate to control cell deformation and migration in microchannels.

Our numerical method is based on the continuum-particle coupling (Akerkouch and Le, 2021), which allows the simulations of RBC dynamics under physiological conditions. Our numerical results show excellent agreements with available *in vitro* and computational data both in cellular mechanics and extracellular flow pattern of the blood plasma(Mills et al, 2004; Guckenberger et al, 2018; Yaya et al, 2021). While most previous studies (Fedosov et al, 2010; Pivkin and Karniadakis, 2008) have only focused on the impact of constant shear rate on the dynamics of the RBCs, our results show that the unsteady shear rate can induce complex RBC’s morphology in confined channels as discussed below.

### 4.1 The emergence of the croissant shape and the slipper shape under a constant shear rate $\overset{-}{{\dot{\gamma }}_{0}}$)

In micro-channel flows with constant shear rate $\left(\overset{-}{{\dot{\gamma }}_{0}}\right)$, three common dynamics of RBCs are frequently observed: (*i*) tumbling; (*ii*) croissant/parachute; and (*iii*) slipper shapes as shown in Figure 5. In unconfined flows (Dupire et al, 2012), the RBC dynamics depends on only the shear rate ($\dot{\gamma }$ or Ca) and viscosity contrast (λ). However, the confinement of micro-channel flows imposes an additional condition for shape transition via the confinement ratio χ. Our results in Figure 5 further confirm that the combination of $C_{a}$ and χ dictates the emergence of RBC shape in either the croissant or slipper shapes.

Recent works (Guckenberger et al, 2018; Yaya et al, 2021) in rectangular microchannels, which are identical to our channels as shown in Figure 2 and Table 4, also suggest that the emergence of RBC shape is dependent on the radial shift (r - see Figure 2 for its definition). In previous works (Guckenberger et al, 2018; Yaya et al, 2021), the croissant shape emerged at $\overset{-}{{\dot{\gamma }}_{0}}<300{\;s}^{-1}$ if the RBC is placed exactly at the channel’s center (r=0). On the other hand, the slipper shape emerged whenever the RBC was not placed exactly at the centerline (r>0). The RBC was found to exhibit a (tank-treading) slipper shape at sufficiently high shear rate $\left(\overset{-}{{\dot{\gamma }}_{0}}\approx 500{\;s}^{-1}\right)$ and off-centered placement (r>0) (Guckenberger et al, 2018; Yaya et al, 2021). In cylindrical micro-channels (Fedosov et al, 2014), similar observations were confirmed albeit at lower shear rates $\left(0<\overset{-}{{\dot{\gamma }}_{0}}<80{\;s}^{-1}\right)$. These works point to the importance of the radial shift in regulating RBC dynamics. Our results in Figure 5 agree with these observations. Our results show the appearance of croissant-to-slipper transition as the Capillary number (and thus $\overset{-}{{\dot{\gamma }}_{0}}$) increases from 0.1 to 0.37 for a confinement of χ=0.65. The croissant shape emerges when the initial position of the RBC is placed exactly at the channel centerline at a sufficiently low shear rate (Ca=0.1). When the shear rate is increased to Ca=0.37, the slipper shape emerges. Furthermore, our model is able to capture the intricate dynamics of the tank-treading motion, which is characterized by the rotation of the membrane at the shear rate of $600{\;s}^{-1}$ as illustrated in Figure 6. Therefore, our results further confirm the importance of the radial shift in the croissant-to-slipper transition.

### 4.2 The impact of time-varying shear rate $\overset{-}{\dot{\gamma }\left(t\right)}$ on RBC shape

When the inflow varies in a stepwise manner as seen in Figure 3, the shear rate $\left(\overset{-}{\dot{\gamma }}\right)$ changes as a function of time $\overset{-}{\dot{\gamma }}(t)$ with distinct forward $\left(T_{f}\right)$ and backward $\left(T_{b}\right)$ time phases. In all cases ($I_{s}\psi_{1}r_{1}\chi_{2}$, $I_{s}\psi_{2}r_{1}\chi_{2}$, $I_{s}\psi_{3}r_{1}\chi_{2}$, $I_{s}\psi_{4}r_{1}\chi_{3}$, $I_{s}\psi_{5}r_{1}\chi_{3}$, and $I_{s}\psi_{6}r_{1}\chi_{3}$), the RBC is placed exactly at the channel axis $\left(r=r_{1}=0\right)$. The shape transitions are accomplished through consistent transient stretching and compression of the membrane. This occurs as the RBC experiences forward and backward flow phases during the flow cycles. The RBC transitions from a discocyte shape toward a croissant shape during its forward propulsion as shown in Figure 6. Although the backward phase induces the buckling of the cellular membrane, the RBC shape remains relatively symmetrical with respect to the channel axis (multilobes) as shown in Figure 6 at the end of $T_{b}$. This is remarkable given that the maximum shear rate during the backward phase can be sufficiently large $\left({\overset{-}{\dot{\gamma }}}_{f}=200{\;s}^{-1}\right)$ but this symmetry is still maintained. Comparing the case $I_{0}U_{3}r_{1}\chi_{1}$ and $I_{0}U_{4}r_{3}\chi_{1}$ in Table 5, our results suggest that the complete break of symmetry (Recktenwald et al, 2022) (slipper shape) is observed only when the radial shift exists (r>0).

When applying different sinusoidal waveforms ($I_{1}$, $I_{2}$, $I_{3}$ and $I_{4}$) shown in Figure 4 , our results show the ubiquitous presence of croissant and slipper shapes across all shear rates (${\overset{-}{\dot{\gamma }}}_{f}=100,\;150$, and $\left.200{\;s}^{-1}\right)$. The slipper shape appears at (t≈0.3T) whenever the RBC is placed off the channel’s axis (r>0) as shown in Tables 9–12. Note that these waveforms are different in terms of the forward $\left(T_{f}\right)$ and backward $\left(T_{b}\right)$ phases, with the backward phase being the shortest in $I_{4}$. As shown in Table 9, the slipper shape is observed even when the waveform is completely symmetric $\left(I_{1}\right)$ given that r>0 as in: (a) $I_{1}U_{1}r_{2}\chi_{1}$; (b) $I_{1}U_{1}r_{3}\chi_{1}$; (c) $I_{1}U_{2}r_{2}\chi_{1}$; (d) $I_{1}U_{2}r_{3}\chi_{1}$; (e) $I_{1}U_{3}r_{2}\chi_{1}$; and (f) $I_{1}U_{3}r_{3}\chi_{1}$. Hence our results indicate that the flow waveform does not affect the emergence of the RBC shape during the forward phase. Instead, the radial shift plays an essential role in this process.

It has been demonstrated (Lanotte et al, 2016) that the presence of the discocyte shape is correlated with weak shear rates $\overset{-}{\gamma_{0}}$. Under ${\overset{-}{\dot{\gamma }}}_{0}<10{\;s}^{-1}$, the RBC typically maintains its discocyte shape with an 80% probability (Lanotte et al, 2016). However, as the shear rate gradually rises $\left(10{\;s}^{-1}\leq {\overset{-}{\dot{\gamma }}}_{0}\leq 400{\;s}^{-1}\right)$ the likelihood of a discocyte shape decreases to 30%. This observation has been validated even when different viscosity ratios (λ) (Mauer et al, 2018) are considered. In our work, the discocyte shape is ubiquitously observed during the second flow cycle across all applied waveforms ($I_{1}$, $I_{2}$, $I_{3}$, and $I_{4}$) and shear rates (${\overset{-}{\dot{\gamma }}}_{f}=100{\;s}^{-1},150\;s^{-1}$, and $\left.200\;s^{-1}\right)$ with (r>0) as shown in Tables 9–12. Therefore, our results further confirm that discocyte is the most common shape with the range shear rate less than $200{\;s}^{-1}$.

In our study, complex shapes evolve from simpler shapes under the influence of time-dependent waveforms. In the experimental work of Lanotte et al (2016) under constant shear rates, stomatocytes shape was observed to dominate the RBC population (65%) when the shear rate was between $10{\;s}^{-1}<{\overset{-}{\dot{\gamma }}}_{0}<400{\;s}^{-1}$. In our study, the elliptical-rim-shaped stomatocytes only emerge under symmetric waveform $\left(I_{1}\right)$ in the case $I_{1}U_{1}r_{1}\chi_{1}$ under the peak shear rate of ${\overset{-}{\dot{\gamma }}}_{f}=100{\;s}^{-1}$. At high constant shear rates $400\;s^{-1}<{\overset{-}{\dot{\gamma }}}_{0}<2,000{\;s}^{-1}$, experimental data (Lanotte et al, 2016) showed that polylobes shape could emerge. This polylobes shape is characterized by a large number of lobes on the RBC surface, known as multilobes, trilobes, and hexalobes. In the current study, polylobes are also observed across all applied waveforms given that the cell is placed initially at the channel axis $\left(r=r_{1}=0\right)$ even at weak shear rates $\left({\overset{-}{\dot{\gamma }}}_{f}\leq 200\;s^{-1}\right)$ as in Figure 8 and Tables 9–12. For example, the multilobes are observed with all waveforms. The trilobes are both observed in the symmetric waveform $\left(I_{1}U_{2}r_{1}\chi_{1}\right.$ and $I_{1}U_{3}r_{1}\chi_{1}$ - Table 9) or the asymmetric waveform $\left(I_{3}U_{2}r_{1}\chi_{1}\right.$ - Table 11). The hexalobes shape only appears under the most asymmetric waveform with $\left(I_{4}U_{2}r_{1}\chi_{1}\right)-{\overset{-}{\dot{\gamma }}}_{f}=150{\;s}^{-1}$ as shown in Table 12. In the previous work of Li et al (2014), the RBC shape has been reported to deform further into elongated shapes as the shear rate increases. Our results support this observation as shown in Figure 11g as the elongated multilobes appear during the backward flow phase. Examining Table 9 to Table 12, our results suggest that this elongated shape is generally present regardless of the applied waveform but it only manifests under higher shear rates of at least ${\overset{-}{\dot{\gamma }}}_{f}=150{\;s}^{-1}$. Specifically, the elongated multilobes are observed under $I_{1}U_{2}r_{1}\chi_{1}$, $I_{1}U_{3}r_{1}\chi_{1}$, $I_{2}U_{3}r_{2}\chi_{1}$, $I_{2}U_{3}r_{3}\chi_{1}$, $I_{3}U_{3}r_{2}\chi_{1}$, and $I_{3}U_{3}r_{3}\chi_{1}$. In essence, our results strongly suggest that the complex shape can appear in a micro-channel even at weak shear rates given that the inflow is time-dependent.

### 4.3. Controlling lateral migration of cells with oscillatory flows

Microfluidic devices are typically used to isolate and separate cells (Gossett et al, 2010). While these devices are promising for many cell-sorting applications (Suresh, 2007; Brandao et al, 2003), the main challenge is the difficulty in obtaining high-throughputs due to the required length of the microfluidic channels. Changing the geometrical design of channels (Amirouche et al, 2020) has been proposed as one efficient way. Recent works have shown that varying the shear rates in time (Schmidt et al, 2022; Krauss et al, 2022) can reduce the required length based on the concept of velocity lift (Qi and Shaqfeh, 2017), which is the factor that drives the RBC’s migration towards the center of the channel.

As the inertial effect is negligible at a very low Reynolds number $\left(R_{e}\approx 0.01\right)$, the flow is reversible for a rigid body. Thus, a rigid body will return to its initial position if the inflow conditions in the backward phase are reversed in the exact opposite way of its own during the forward phase. However, the RBC is not a rigid body and its membrane is highly flexible. Experiments by Krauss et al (2022) have shown that the responses of RBCs are different from the ones of stiff beads in oscillatory flows. The RBC migration is observed to have a net actuation in oscillating flows whereas a stiff bead does not.

Our results in Figures 10 and 9 for the symmetric waveform $\left(I_{1}\right)$ show that the RBC does not go back to its initial position at the end of the cycle. There is an axial shift of the RBC from its original position $\left(\Delta x_{c}\neq 0\right)$ at the end of the cycle. Moreover, the RBC migrates significantly in the lateral cross-section $\left(\Delta y_{c}\gg 0\right.$ and $\left(\Delta z_{c}\gg 0\right)$ as shown in Figure 9. Our results in Figures 10b and 10e indicate that the RBC undergoes lateral migration. This migration is particularly evident in the cases $I_{1}U_{1}r_{3}\chi_{1}$ and $I_{4}U_{1}r_{3}\chi_{1}$, where the cell migrates in the lateral direction for a distance of approximately $0.25L_{s}$ and $0.13L_{s}$, respectively. Comparing the shapes of the waveform $I_{1}$, $I_{2}$, $I_{3}$, and $I_{4}$ in Figure 4, our results suggest that significant lateral migration can be induced by adjusting the forward time interval $\left(T_{f}\right)$. With our sinusoidal waveforms in Figure 4, $T_{f}$ should be less than three times the backward flow phase $T_{b}(\frac{T_{f}}{T_{b}}<3.)$ to be able to induce significant lateral migration. This lateral migration in oscillatory flows might be used to separate cells selectively based on their mechanical properties.

Our findings in Figure 7 and Figure 11 show that the extracellular flow patterns are directly influenced by the dynamics of the RBC. Under a stationary condition in Figure 7, the extracellular flow dynamics in the croissant shape is distinctively different from the ones of the slipper shape. In particular, the flow around the steady croissant shape was found similar to that of a rigid sphere (Lee et al, 2010), in which the flow streamlines move nearly symmetrically inwards and outward from the cell in the upstream and downstream sides, respectively. In contrast, a fully-closed vortex ring (“bolus”) was observed downstream the cell for the slipper shape(Guckenberger et al, 2018). Our results in Figure 11 suggest that the extracellular flow patterns are far more complicated and highly dependent on the type of inflow waveform in time-dependent shear rates. It is important to note that the extracellular flow has been found to play an important role in drug delivery strategies(Yaya et al, 2021) due to its potential use of particle trapping. Therefore, our results suggest that controlling the inflow waveform either by adjusting the peak flow $\psi_{f}$ or the shape of the waveform (specifically $T_{f}$) might lead to the desired effects in delivering small particles (e.x therapeutic nano-particles) to the RBCs.

## 5 Conclusion

Transient dynamics of Red Blood Cells (RBC) in confined channels under oscillatory flows are investigated using our continuum-particle approach (Akerkouch and Le, 2021). Our results reveal that the dynamics of RBCs are complex with different shape modes that are beyond the usually observed croissant and slipper modes. Our results indicate that the extracellular flow pattern around the RBC is dependent on the RBC shape. Our results suggest that the oscillatory flow can be used to control and manipulate the dynamics of RBC by adapting the appropriate flow waveform. Our specific conclusions are:

- The RBC can transform into a variety of shapes such as multilobes, trilobes and hexalobes by varying the sinusoidal waveform even when it is subjected to a relatively weak shear rate $\left(\overset{-}{{\dot{\gamma }}_{f}}\leq 200\;s^{-1}\right)$.
- Simple shapes such as croissant, slipper, and rolling discocyte appear when the RBC is subjected to all waveforms. However, complex shapes such as rolling stomatocytes, trilobes, and hexalobes appeared only under specific conditions. In our study, the RBC transitions into 8 shapes under the symmetric waveform $\left(I_{1}\right)$, and into 5 shapes under the asymmetric waveform $\left(I_{2}\right)$. Therefore, it is possible to attain a certain shape using an appropriate waveform.
- Under the symmetric flow waveform, the axial displacement of the RBC is rather minimal. However, the lateral displacements are significantly large. Under the asymmetric flow waveform, the RBC experiences a large axial displacement but small lateral displacements.
- The maximum lateral displacement of the RBC during its propagation depends on the initial radial shift (r). This maximum value is also dependent on the asymmetry of the flow waveform (I).
- The extracellular flow surrounding the RBC depends on its morphological shape. The flow pattern is thus distinct and unique for each shape.

## Figures and Tables

**Table 1:** The relationship between DPD parameters and the physical units for viscosity ratio $\lambda =5.N_{m},\;m,\;\delta t$ and $\mu_{cytosol}$ correspond to the number of molecules in one bead, mass, time step and dynamic viscosity of the cytosol, respectively. V is the volume of the water molecule (30 *Å*), M is the molar weight of water (18 *g mol*^−1^) and $N_{A}=6.0221415\times 10^{23}$ is the Avogardo’s constant. The definitions of the parameters $k_{B}T$, γ, ρ and $r_{c}$ are explained in Table 2 and section 2.3.4.

Parameters	DPD value	Physical unit	Physical value

Bead	1	$N_{m}$	$3H_{2}O$
$r_{c}$	1	${\left(\rho N_{m}V\right)}^{\frac{1}{3}}$	6.45 *Å*
m	1	$\frac{N_{m}M}{N_{A}}$	8.98 × 10^−23^ *g*
ρ	3	$\frac{\rho N_{m}M}{N_{A}r_{c}^{3}}$	996.3 *kg m*^−3^
δt	0.01	$\tau :\delta tr_{c}\sqrt{\frac{m}{k_{B}T}}$	1 *ps*
$\mu_{cytosol\;}(\gamma =116.4)$	4.11 ± 0.1	$\frac{\eta \tau k_{B}T}{r_{c}^{3}}$	0.006 *Pa s*

**Table 2:** The physical parameters describing the RBC characteristics

RBC physical parameters	

RBC diameter $\left(D_{0}\right)$	7.82 *μm*
RBC area $\left(A_{0}^{tot}\right)$	135.0 × 10^−12^ *m*^2^
RBC volume $\left(V_{0}^{tot}\right)$	94.0 × 10^−18^ *m*^3^
Elastic shear modulus $\left(\mu_{0}\right)$	6.3 *μN/m*
Young’s modulus (Y)	18.9 *μN/m*
Bending rigidity $\left(k_{b}\right)$	3.0 × 10^−19^ *J*
Membrane viscosity $\left(\eta_{m}\right)$	22.0 × 10^−3^ *Pa s*
Boltzmann’s constant $\left(k_{B}\right)$	1.380649 × 10^−23^ *m*^2^ *kg s*^−2^ *K*^−1^
Temperature (T)	298 *K*

**Table 3:** Coarse-grained parameters for the RBC membrane model for different numbers of vertices $N_{v}$. The definitions of the parameters $D_{0}^{M}$, $l_{0}$, $l_{max}$, p, $k_{p}$ and $\theta_{0}$ are explained in sections 2.2 and 2.4. The corresponding values of Young’s modulus, global area, local area and volume constraints in DPD units are $Y^{M}=392.5$, $k_{a}=4900$, $k_{d}=100$, $k_{v}=5000$, respectively. Other parameters are α=1 and $\eta_{m}^{M}=1.8$.

$N_{v}$	$D_{0}^{M}$	$l_{0}(m)$	$l_{max}(m)$	p(m)	$k_{p}\left(N{\;m}^{2}\right)$	$\theta_{0}(deg)$

500	8.07	5.5614 × 10^−7^	1.2235 × 10^−6^	1.9933 × 10^−9^	6.6288 × 10^−25^	6.86
1000	8.07	3.7992 × 10^−7^	8.3582 × 10^−7^	2.9179 × 10^−9^	2.1132 × 10^−25^	4.69
3000	8.07	2.2818 × 10^−7^	5.0199 × 10^−7^	4.8584 × 10^−9^	4.5783 × 10^−26^	2.82
9000	8.07	1.3035 × 10^−7^	2.8678 × 10^−7^	8.5044 × 10^−9^	8.5357 × 10^−27^	1.61
24, 472	8.26	7.5331 × 10^−8^	1.6573 × 10^−7^	1.4716 × 10^−8^	1.6474 × 10^−27^	0.93

**Table 4:** Different channel geometries and their associated computational grids to simulate the dynamics of RBC in fluid flows. The channels have rectangular cross-sections of size $L_{x}$, $L_{y}$, and $L_{z}$ along the axial, spanwise, and vertical directions, respectively. $N_{i}$, $N_{j}$ and $N_{k}$ are respectively the number of grid points in x, y, z directions. χ is the channel confinement, which is defined in section 2.8.

Channel	$L_{x}\times L_{y}\times L_{z}(\mu m)$	$N_{i}\times N_{j}\times N_{k}$	Δx×Δy×Δz(μm)	χ

1	90 × 12 × 10	151 × 101 × 101	0.6 × 0.12 × 0.1	0.65
2	80 × 16 × 16	151 × 101 × 101	0.54 × 0.16 × 0.16	0.4
3	80 × 21 × 21	151 × 151 × 151	0.54 × 0.14 × 0.14	0.3

**Table 5:** Summary of the validation cases under constant shear rates $\left(I_{0}U_{3}r_{1}\chi_{1}\right.$ and $\left.I_{0}U_{4}r_{3}\chi_{1}\right)$, and stepwise oscillatory flows ($I_{s}\psi_{1}r_{1}\chi_{2}$, $I_{s}\psi_{2}r_{1}\chi_{2}$, $I_{s}\psi_{3}r_{1}\chi_{2}$ and $I_{s}\psi_{4}r_{1}\chi_{3}$, $I_{s}\psi_{5}r_{1}\chi_{3}$, $I_{s}\psi_{6}r_{1}\chi_{3}$). The stepwise oscillatory flows with the forward $\left(\psi_{f}\right)$, backward $\left(\psi_{b}\right)$ velocities and the forward Capillary number are defined in section 2.8.2. The maximum shear rate $\left({\overset{-}{\dot{\gamma }}}_{f}\right)$ and the maximum Reynolds number $\left(Re_{f}\right)$ are defined in section 2.8.1. The definition of the RBC’s radial shift r is shown in Figure 2b.

Case	Inflow	$\psi_{f}(\;mm/s)$	$\psi_{b}(\;mm/s)$	${\overset{-}{\dot{\gamma }}}_{f}\left({\;s}^{-1}\right)$	r(μm)	$Re_{f}$	$Ca^{f}$

$I_{0}U_{3}r_{1}\chi_{1}$	$I_{0}$	2	-	200	0	1.34 × 10^−2^	0.49
$I_{0}U_{4}r_{3}\chi_{1}$	$I_{0}$	6	-	600	0.7	4 × 10^−2^	1.47
$I_{s}\psi_{1}r_{1}\chi_{2}$	$I_{s}$	1.05	−0.27	66	0	7 × 10^−3^	0.1
$I_{s}\psi_{1}r_{1}\chi_{2}$	$I_{s}$	1.58	−0.39	98	0	1.05 × 10^−2^	0.15
$I_{s}\psi_{2}r_{1}\chi_{2}$	$I_{s}$	2.1	−0.53	132	0	1.4 × 10^−2^	0.2
$I_{s}\psi_{4}r_{1}\chi_{3}$	$I_{s}$	1.9	−0.48	119	0	1.27 × 10^−2^	0.1
$I_{s}\psi_{5}r_{1}\chi_{3}$	$I_{s}$	2.8	−0.7	175	0	1.87 × 10^−2^	0.15
$I_{s}\psi_{6}r_{1}\chi_{3}$	$I_{s}$	3.7	−0.93	232	0	2.47 × 10^−2^	0.2

**Table 6:** The controlling parameters of the pulsatile waveforms ($I_{1}$, $I_{2}$, $I_{3}$ and $I_{4}$). The waveforms are characterized by the intervals of the forward $\left(T_{f}\right)$, rest $\left(T_{r}\right)$ and backward $\left(T_{b}\right)$ periods. The shapes of the waveforms are shown in Figure 4.

_Waveforms_╲^Time^	$T_{f}\;(ms)$	$T_{r}\;(ms)$	$T_{b}\;(ms)$

$I_{1}$	20	10	20
$I_{2}$	25	12.5	12.5
$I_{3}$	27.3	9	13.7
$I_{4}$	28.6	7.1	14.3

**Table 7:** The summary of the combinatoric configurations for steady and pulsatile flow simulations. The combination of the waveform type, the forward flow velocity, the radial shift, and the channel confinement results in the simulation configurations of $I_{m}U_{n}r_{p}\chi_{s}$. Here m=1,2,3, and 4, n=1,2,3 and 4, p=1,2 and 3 and s=1,2 and 3. The profile of the inflow waveforms ($I_{1}$, $I_{2}$, $I_{3}$ and $I_{4}$) are shown in Figure 4. The peak forward flow velocity *U* (Figure 4a) varies from 1 to 6 mm/s. The radial shift of the RBC centroid along the bisector of the y-z plane at the initial time is defined in Figure 2b.

Subscript	Inflow waveform	$\psi_{f}(\;mm/s)$	Radial shift r(μm)

1	$I_{1}$	$U_{1}=1$	$r_{1}=0$
2	$I_{2}$	$U_{2}=1.5$	$r_{2}=0.4$
3	$I_{3}$	$U_{3}=2$	$r_{3}=0.7$
4	$I_{4}$	$U_{4}=6$	–

**Table 8:** Summary of the 36 sinusoidal flow cases in section 2.8.3 with a confinement of $\chi_{1}=0.65$. Tables (*a*), (*b*), (*c*), or (*d*) each consist of 9 possible combinations between the peak forward flow U and the radial shift r for each type of waveform $I_{1}$, $I_{2}$, $I_{3}$ and $I_{4}$, respectively. The exact numeric value of $U_{1}$, $U_{2}$, $U_{3}$ and $r_{1}$, $r_{2}$, $r_{3}$ are shown in Table 7.

(a)					(b)				
		$r_{1}$	$r_{2}$	$r_{3}$			$r_{1}$	$r_{2}$	$r_{3}$
$I_{1}$	$U_{1}$	$I_{1}U_{1}r_{1}\chi_{1}$	$I_{1}U_{1}r_{2}\chi_{1}$	$I_{1}U_{1}r_{3}\chi_{1}$	$I_{2}$	$U_{1}$	$I_{2}U_{1}r_{1}\chi_{1}$	$I_{2}U_{1}r_{2}\chi_{1}$	$I_{2}U_{1}r_{3}\chi_{1}$
	$U_{2}$	$I_{1}U_{2}r_{1}\chi_{1}$	$I_{1}U_{2}r_{2}\chi_{1}$	$I_{1}U_{2}r_{3}\chi_{1}$		$U_{2}$	$I_{2}U_{2}r_{1}\chi_{1}$	$I_{2}U_{2}r_{2}\chi_{1}$	$I_{2}U_{2}r_{3}\chi_{1}$
	$U_{3}$	$I_{1}U_{3}r_{1}\chi_{1}$	$I_{1}U_{3}r_{2}\chi_{1}$	$I_{1}U_{3}r_{3}\chi_{1}$		$U_{3}$	$I_{2}U_{3}r_{1}\chi_{1}$	$I_{2}U_{3}r_{2}\chi_{1}$	$I_{2}U_{3}r_{3}\chi_{1}$
(c)					(d)				
		$r_{1}$	$r_{2}$	$r_{3}$			$r_{1}$	$r_{2}$	$r_{3}$
$I_{3}$	$U_{1}$	$I_{3}U_{1}r_{1}\chi_{1}$	$I_{3}U_{1}r_{2}\chi_{1}$	$I_{3}U_{1}r_{3}\chi_{1}$	$I_{4}$	$U_{1}$	$I_{4}U_{1}r_{1}\chi_{1}$	$I_{4}U_{1}r_{2}\chi_{1}$	$I_{4}U_{1}r_{3}\chi_{1}$
	$U_{2}$	$I_{3}U_{2}r_{1}\chi_{1}$	$I_{3}U_{2}r_{2}\chi_{1}$	$I_{3}U_{2}r_{3}\chi_{1}$		$U_{2}$	$I_{4}U_{2}r_{1}\chi_{1}$	$I_{4}U_{2}r_{2}\chi_{1}$	$I_{4}U_{2}r_{3}\chi_{1}$
	$U_{3}$	$I_{3}U_{3}r_{1}\chi_{1}$	$I_{3}U_{3}r_{2}\chi_{1}$	$I_{3}U_{3}r_{3}\chi_{1}$		$U_{3}$	$I_{4}U_{3}r_{1}\chi_{1}$	$I_{4}U_{3}r_{2}\chi_{1}$	$I_{4}U_{3}r_{3}\chi_{1}$

**Table 9:** Summary of the RBC morphology transition sequences recorded at different time instances $\frac{t}{T}$ under $I_{1}$ waveform, and different flow velocities $U_{1}$, $U_{2}$, $U_{3}$ and radial shift $r_{1}$, $r_{2}$, $r_{3}$. Here, the time instances represent the first time the RBC deformed shape appeared. The acronyms C, S, CM, M, T, RS, EM and RD represent the croissant, slipper, complex multilobes, multilobes, trilobes, rolling stomatocytes, elongated multilobes and rolling discocyte, respectively. The exact numeric value of $U_{1}$, $U_{2}$, $U_{3}$ and $r_{1}$, $r_{2}$, $r_{3}$ are shown in Table 7.

Waveform $\left(I_{1}\right)$	C	S		CM	M	T	RS	EM	RD		
							
	$r_{1}$	$r_{2}$	$r_{3}$	$r_{1}$	$r_{1}$	$r_{1}$	$r_{1}$	$r_{1}$	$r_{1}$	$r_{2}$	$r_{3}$

$U_{1}$	0.32	0.3	0.3	-	0.75	-	0.9	-	1.8	1.25	1.25
$U_{2}$	0.21	0.2	0.2	0.5	-	0.8	-	1.2	-	1.21	1.21
$U_{3}$	0.21	0.2	0.2	0.5	-	0.8	-	1.2	-	1.21	1.21

**Table 10:** Summary of the RBC morphology transition sequences recorded at different time instances $\frac{t}{T}$ under $I_{2}$ waveform, and the different flow velocities $U_{1}$, $U_{2}$, $U_{3}$ and initial placements $r_{1}$, $r_{2}$, $r_{3}$. Here, the time instances represent the first time the RBC deformed shape appeared. The acronyms C, S, CM, EM and RD represent the croissant, slipper, complex multilobes, elongated multilobes and rolling discocyte, respectively. The exact numeric value of $U_{1}$, $U_{2}$, $U_{3}$ and $r_{1}$, $r_{2}$, $r_{3}$ are shown in Table 7.

Waveform $\left(I_{2}\right)$	C	S		CM	EM		RD		
				
	$r_{1}$	$r_{2}$	$r_{3}$	$r_{1}$	$r_{2}$	$r_{3}$	$r_{1}$	$r_{2}$	$r_{3}$

$U_{1}$	0.27	0.29	0.29	0.9	-	-	1.55	1.33	1.33
$U_{2}$	0.2	0.22	0.22	1.06	-	-	1.44	1.3	1.3
$U_{3}$	0.2	0.22	0.22	1.06	1.16	1.16	1.8	1.3	1.3

**Table 11:** Summary of the RBC morphology transition sequences recorded at different time instances $\frac{t}{T}$ under $I_{3}$ waveform, and the different flow velocities $U_{1}$, $U_{2}$, $U_{3}$ and initial placements $r_{1}$, $r_{2}$, $r_{3}$. Here, the time instances represent the first time the RBC deformed shape appeared. The acronyms C, S, CM, T, EM and RD represent the croissant, slipper, complex multilobes, trilobes, elongated multilobes and rolling discocyte, respectively. The exact numeric value of $U_{1}$, $U_{2}$, $U_{3}$ and $r_{1}$, $r_{2}$, $r_{3}$ are shown in Table 7.

Waveform $\left(I_{3}\right)$	C	S		CM	T	EM		RD		
					
	$r_{1}$	$r_{2}$	$r_{3}$	$r_{1}$	$r_{1}$	$r_{2}$	$r_{3}$	$r_{1}$	$r_{2}$	$r_{3}$

$U_{1}$	0.34	0.27	0.27	0.58	-	-	-	1.35	1.35	1.35
$U_{2}$	0.28	0.22	0.22	0.62	0.94	-	-	1.8	1.32	1.32
$U_{3}$	0.28	0.22	0.22	0.62	-	1.18	1.18	1.4	1.32	1.32

**Table 12:** Summary of the RBC morphology transition sequences recorded at different time instances $\frac{t}{T}$ under $I_{4}$ waveform, and the different flow velocities $U_{1}$, $U_{2}$, $U_{3}$ and initial placements $r_{1}$, $r_{2}$, $r_{3}$. Here, the time instances represent the first time the RBC deformed shape appeared. The acronyms C, S, CM, EM, RD and HX represent the croissant, slipper, complex multilobes, elongated multilobes, rolling discocyte and hexalobes, respectively. The exact numeric value of $U_{1}$, $U_{2}$, $U_{3}$ and $r_{1}$, $r_{2}$, $r_{3}$ are shown in Table 7.

Waveform $\left(I_{4}\right)$	C	S		CM	EM		RD			HX
					
	$r_{1}$	$r_{2}$	$r_{3}$	$r_{1}$	$r_{2}$	$r_{3}$	$r_{1}$	$r_{2}$	$r_{3}$	$r_{1}$

$U_{1}$	0.32	0.31	0.31	0.6	-	-	1.38	1.27	1.27	-
$U_{2}$	0.27	0.24	0.24	0.56	-	-	1.5	1.24	1.24	1.15
$U_{3}$	0.27	0.24	0.24	0.56	1.15	1.15	1.1	1.24	1.24	-

## References

[R1] Agarwal D, Biros G (2022) Shape dynamics of a red blood cell in poiseuille flow. Phys Rev Fluid 7(093602)

[R2] Akerkouch L, Le T (2021) A hybrid continuum-particle approach for fluid-structure interaction simulation of red blood cells in fluid flows. Fluids 6(4):139

[R3] Amirouche A, Esteves J, Lavoignat A, (2020) Dual shape recovery of red blood cells flowing out of a microfluidic constriction. Biomicrofluidics 14(2) 10.1063/5.0005198 PMC7190370 32549922

[R4] Barabino G, Platt M, Kaul D (2010) Sickle cell biomechanics. Annu Rev Biomed Eng 12:345–367 20455701 10.1146/annurev-bioeng-070909-105339

[R5] Brandao M, Fontes A, Barjas-Castro M, (2003) Optical tweezers for measuring red blood cell elasticity: Application to the study of drug response in sickle cell disease. Eur J Haematol 70(207) 10.1034/j.1600-0609.2003.00027.x 12656742

[R6] Coupier G, Farutin A, Minetti C, (2012) Shape diagram of vesicles in poiseuille flow. Physical review letters 108(17):178106 22680911 10.1103/PhysRevLett.108.178106

[R7] Czaja B, Gutierrez M, Zavodszky G, (2020) The influence of red blood cell deformability on hematocrit profiles and platelet margination. PLoS Comput Biol 16(3):e1007716 32163405 10.1371/journal.pcbi.1007716 PMC7093031

[R8] Dupire J, Socol M, Viallat A (2012) Full dynamics of a red blood cell in shear flow. Proceedings of the National Academy of Sciences 109(51):20808–20813 10.1073/pnas.1210236109 PMC3529085 23213229

[R9] Fan X, Phan-Thien N, Chen S, (2006) Simulating flow of dna suspension using dissipative particle dynamics. Phys Fluids 18(063102)

[R10] Fedosov D, Peltomaki M, Gompper G (2014) Deformation and dynamics of red blood cells in flow through cylindrical microchannels. Soft Matter 10:4258–4267 24752231 10.1039/c4sm00248b

[R11] Fedosov F, Caswell B, Karniadakis G (2010) A multiscale red blood cell model with accurate mechanics, rheology, and dynamics. Biophys J 98:2215–2225 20483330 10.1016/j.bpj.2010.02.002 PMC2872218

[R12] Ge L, Sotiropoulos F (2007) A numerical method for solving the 3d unsteady incompressible navier-stokes equations in curvilinear domains with complex immersed boundaries. J Comput Phys 225(2):1782 19194533 10.1016/j.jcp.2007.02.017 PMC2635106

[R13] Ghoufi A, Emile J, Malfreyt P (2013) Recent advances in many body dissipative particles dynamics simulations of liquid-vapor interfaces. Eur Phys J E 36(10) 10.1140/epje/i2013-13010-7 23361618

[R14] Gossett D, Weaver W, Mach A, (2010) Label-free cell separation and sorting in microfluidic systems. Analytical and bioanalytical chemistry 397(8):3249–3267 20419490 10.1007/s00216-010-3721-9 PMC2911537

[R15] Groot R, Warren P (1997) Dissipative particle dynamics: Bridging the gap between atomistic and mesoscopic simulation. J Chem Phys 107(11)

[R16] Guckenberger A, Kihm A, John T, (2018) Numerical-experimental observation of shape bistability of red blood cells flowing in a microchannel. Soft Matter 14:2032–2043 29473072 10.1039/c7sm02272g

[R17] Kaoui B, Biros G, Misbah C (2009) Why do red blood cells have asymmetric shapes even in a symmetric flow? Physical review letters 103(18):188101 19905834 10.1103/PhysRevLett.103.188101

[R18] Kaoui B, Harting J, Misbah C (2011a) Two-dimensional vesicle dynamics under shear flow: Effect of confinement. Physical Review E 83(6):066319 10.1103/PhysRevE.83.066319 21797489

[R19] Kaoui B, Tahiri T, Biben T, (2011b) Complexity of vesicle microcirculation. Phys Rev E: Stat, Nonlinear, Soft Matter Phys 84:041906 10.1103/PhysRevE.84.041906 22181174

[R20] Kaul D, Fabry M, Windisch P, (1983) Erythrocytes in sickle cell anemia are heterogeneous in their rheological and hemodynamic characteristics. J Clin Invest 72:22–31 6874947 10.1172/JCI110960 PMC1129157

[R21] Kihm A, Kaestner L, Wagner C, (2018) Classification of red blood cell shapes in flow using outlier tolerant machine learning. PLoS Comput Biol 14(e1006278) 10.1371/journal.pcbi.1006278 PMC6021115 29906283

[R22] Krauss S, Gires P, Weiss M (2022) Deformation-induced actuation of cells in asymmetric periodic flow fields. Phys Rev fluids 7(L082201)

[R23] Lafzi A, Raffiee A, Dabiri S (2020) Inertial migration of a deformable capsule in an oscillatory flow in a microchannel. Physical Review E 102(6):063110 33466115 10.1103/PhysRevE.102.063110

[R24] Lanotte L, Mauer J, Mendez S, (2016) Red cells’ dynamic morphologies govern blood shear thinning under microcirculatory flow conditions. PNAS 13(47):13289–13294 10.1073/pnas.1608074113 PMC5127344 27834220

[R25] Le T, Sotiropoulos F (2012) On the three-dimensional vortical structure of early diastolic flow in a patient-specific left ventricle. Eur J Mech B Fluids 35:20–24 22773898 10.1016/j.euromechflu.2012.01.013 PMC3388554

[R26] Le T, Borazjani I, Sotiropoulos F (2010) Pulsatile flow effects on the hemodynamics of intracranial aneurysms. J Biomech Eng 132(11):111009 21034150 10.1115/1.4002702

[R27] Le T, Elbaz M, Geest RVD, (2019) High resolution simulation of diastolic left ventricular hemodynamics guided by four-dimensional flow magnetic resonance imaging data. Flow Turbul Combust 102(1):3–26

[R28] Lee W, Aminia H, Stonec H, (2010) Dynamic self-assembly and controlof microfluidic particle crystals. PNAS 107(52):22413–22418 21149674 10.1073/pnas.1010297107 PMC3012521

[R29] Li X, Peng Z, Lei H, (2014) Probing red blood cell mechanics, rheology and dynamics with a two-component multi-scale model. Phil Trans R Soc A 372(20130389) 10.1098/rsta.2013.0389 PMC4084529 24982252

[R30] Li X, Lu H, Peng Z (2018) Continuum and particle-based modeling of human red blood cells. Handbook of Materials Modeling Applications: Current and Emerging Materials

[R31] Mai-Duy N, Phan-Thien N, Nguyen T, (2020) Coarse-graining, compressibility, and thermal fluctuation scaling in dissipative particle dynamics employed with pre-determined input parameters. Phys Fluids 32:053313

[R32] Mauer J, Mendez S, Lanotte L, (2018) Flow-induced transitions of red blood cell shapes under shear. Phys Rev Lett 121(118103) 10.1103/PhysRevLett.121.118103 30265089

[R33] McWhirter J, Noguchi H, Gompper G (2009) Flow-induced clustering and alignment of vesicles and red blood cells in microcapillaries. PNAS 106(15):6039–43 19369212 10.1073/pnas.0811484106 PMC2669370

[R34] Mills J, Qie L, Dao M, (2004) Nonlinear elastic and viscoelastic deformation of the human red blood cell with optical tweezers. Mech Chem Biosyst 1:169–180 16783930

[R35] Mutlu B, Edd J, Toner M (2018) Oscillatory inertial focusing in infinite microchannels. Proceedings of the National Academy of Sciences 115(30):7682–7687 10.1073/pnas.1721420115 PMC6065022 29991599

[R36] Noguchi† H, Gompper G (2005) Shape transitions of fluid vesicles and red blood cells in capillary flows. PNAS 102(40):14159–14164 16186506 10.1073/pnas.0504243102 PMC1242298

[R37] Peng Z, Li X, Pivkin I, (2013a) Lipid bilayer and cytoskeletal interactions in a red blood cell. PNAS 110(33):13356–13361 23898181 10.1073/pnas.1311827110 PMC3746879

[R38] Peng Z, Li X, Pivkin I, (2013b) Lipid bilayer and cytoskeletal interactions in a red blood cell. Proceedings of the National Academy of Sciences 110(33):13356–13361 10.1073/pnas.1311827110 PMC3746879 23898181

[R39] Pivkin I, Karniadakis G (2008) Accurate coarse-grained modeling of red blood cells. PRL 101(11):118105 10.1103/PhysRevLett.101.118105 18851338

[R40] Prado G, Farutin A, Misbah C, (2015) Viscoelastic transient of confined red blood cells. Biophys J 108(9):2126–2136 25954871 10.1016/j.bpj.2015.03.046 PMC4423063

[R41] Qi Q, Shaqfeh E (2017) Theory to predict particle migration and margination in the pressure-driven channel flow of blood. Phys Rev Fluids 2(093102)

[R42] Quint S, Christ A, Guckenberger A, (2017) 3d tomography of cells in micro-channels. Appl Phys Lett 111(103701)

[R43] Recktenwald S, Graessel K, Maurer F, (2022) Red blood cell shape transitions and dynamics in time-dependent capillary flows. Biophysical Journal 121(1):23–36 34896369 10.1016/j.bpj.2021.12.009 PMC8758421

[R44] Reichel F, Mauer J, Nawaz A, (2019) High-throughput microfluidic characterization of erythrocyte shapes and mechanical variability. Biophys J 107:14–24 10.1016/j.bpj.2019.05.022 PMC6626834 31235179

[R45] Schmidt W, Fortsch A, Laumann M, (2022) Oscillating non-progressing flows induce directed cell motion. Physical Review Fluids 7(3):L032201

[R46] Secomb T (2017) Blood flow in the microcirculation. Annual Review of Fluid Mechanics 49:443–461

[R47] Suresh S (2007) Biomechanics and biophysics of cancer cells. Acta Biomater 3(413) 10.1016/j.actbio.2007.04.002 PMC2917191 17540628

[R48] Tang Y, Lu L, Li H, (2017) Openrbc: A fast simulator of red blood cells at protein resolution. Biophysical journal 112(10):2030–2037 28538143 10.1016/j.bpj.2017.04.020 PMC5444005

[R49] Tomaiulo G, Simeone M, Martinelli V, (2009) Red blood cell deformation in microconfined flow. Soft Matter 5(3736)

[R50] Vlahovska P, Young Y, Danker G, (2011) Dynamics of a non-spherical micro-capsule with incompressible interface in shear flow. Journal of fluid mechanics 678:221–247

[R51] Wells R, Schmid-Schönbein H (1969) Red cell deformation and fluidity of concentrated cell suspensions. J Appl Physiol 27(2):213–7 5796309 10.1152/jappl.1969.27.2.213

[R52] Yaya F, Romer J, Guckenberger A, (2021) Vortical flow structures induced by red blood cells in capillaries. Microcirculation 28(5) 10.1111/micc.12693 33666310

[R53] Ye T, Pan D, Huang C, (2019) Smoothed particle hydrodynamics (sph) for complex fluid flows: Recent developments in methodology and applications. Physics of Fluids 31(1):011301. 10.1063/1.5068697, URL https://doi.org/10.1063/1.5068697,

